# Signaling dynamics in coexisting monoclonal cell subpopulations unveil mechanisms of resistance to anti-cancer compounds

**DOI:** 10.1186/s12964-024-01742-3

**Published:** 2024-07-26

**Authors:** Claire E. Blanchard, Alison T. Gomeiz, Kyle Avery, Emna El Gazzah, Abduljalil M. Alsubaie, Masoumeh Sikaroodi, Ylenia Chiari, Chelsea Ward, Jonathan Sanchez, Virginia Espina, Emanuel Petricoin, Elisa Baldelli, Mariaelena Pierobon

**Affiliations:** 1 School of Systems Biology, George Mason University, 10920 George Mason Circle, Room 2016, Manassas, VA 20110 USA; 2 Microbiome Analysis Center, George Mason University, Manassas, VA 20110 USA; 3 Department of Biology, George Mason University, Fairfax, VA 22030 USA; 4 School of Life Sciences, University of Nottingham, Nottingham, NG7 2TQ UK; 5 Center for Applied Proteomics and Molecular Medicine, George Mason University, Manassas, VA 20110 USA

## Abstract

**Background:**

Tumor heterogeneity is a main contributor of resistance to anti-cancer targeted agents though it has proven difficult to study. Unfortunately, model systems to functionally characterize and mechanistically study dynamic responses to treatment across coexisting subpopulations of cancer cells remain a missing need in oncology.

**Methods:**

Using single cell cloning and expansion techniques, we established monoclonal cell subpopulations (MCPs) from a commercially available epidermal growth factor receptor (EGFR)-mutant non-small cell lung cancer cell line. We then used this model sensitivity to the EGFR inhibitor osimertinib across coexisting cell populations within the same tumor. Pathway-centered signaling dynamics associated with response to treatment and morphological characteristics of the MCPs were assessed using Reverse Phase Protein Microarray. Signaling nodes differentially activated in MCPs less sensitive to treatment were then pharmacologically inhibited to identify target signaling proteins putatively implicated in promoting drug resistance.

**Results:**

MCPs demonstrated highly heterogeneous sensitivities to osimertinib. Cell viability after treatment increased > 20% compared to the parental line in selected MCPs, whereas viability decreased by 75% in other MCPs. Reduced treatment response was detected in MCPs with higher proliferation rates, EGFR L858R expression, activation of EGFR binding partners and downstream signaling molecules, and expression of epithelial-to-mesenchymal transition markers. Levels of activation of EGFR binding partners and MCPs’ proliferation rates were also associated with response to c-MET and IGFR inhibitors.

**Conclusions:**

MCPs represent a suitable model system to characterize heterogeneous biomolecular behaviors in preclinical studies and identify and functionally test biological mechanisms associated with resistance to targeted therapeutics.

**Supplementary Information:**

The online version contains supplementary material available at 10.1186/s12964-024-01742-3.

## Background

Whether driven by the expansion of pre-existing clones or the acquisition of new traits, intra- and inter-tumor heterogeneity are main contributors to the development of innate and acquired resistance to anti-cancer targeted agents [1, 2]. Subpopulations of cancer cells adapt to a variety of endogenous and exogenous factors, including treatment, through diverse and often coexisting biological mechanisms [3]. A number of studies have shown that adaptation to treatment-associated selective pressure can be driven by on- and off-target events [4–7]. As a result, significant efforts have been invested to better understand the degree of heterogeneity within and between tumors as well as its effect on response to targeted agents [7–10]. While capturing the genomic landscape of tumors is routinely performed through the collection of tissue biopsies or blood sampling to inform treatment selection, functionally characterizing and understanding the role of coexistent mechanisms of resistance within the same tumor continues to present technical challenges. The collection of tumor biopsies as part of standard clinical practice captures a collective single snapshot of the highly variable molecular landscape within a tumor and is poorly suited for capturing dynamic and heterogeneous cellular behavior [11–13]. Techniques that capture tumor heterogeneity like single-cell sequencing, serial functional imaging, blood sampling, and multi-regional sequencing are being assessed in ongoing clinical trials for their ability to better inform treatment selection in various cancer types [14]. However, they find limited applicability in functionally understanding how individual clones respond or adapt to treatment [15]. To fill this gap, we established monoclonal cell populations (MCPs) from a commercially available non-small cell lung cancer (NSCLC) cell line. We then tested the feasibility of using this model system to capture signaling dynamics associated with response to targeted treatment across distinct, but coexisting, cell subpopulations [16].

Agents targeting the epidermal growth factor receptor (EGFR) have been instrumental pioneers in the field of precision medicine for lung cancer. First- and second-generation EGFR inhibitors designed to target aberrant EGFR activity have significantly impacted outcomes for NSCLC patients whose tumors harbored gain-of-function oncogenic alterations of the receptor gene. However, over time, administration of these compounds inevitably leads to acquired resistance. On-target secondary genomic alterations, like T790M mutations, have been observed in 50–60% of NSCLC patients treated with first- or second-line EGFR tyrosine kinase inhibitors (TKIs) and have been linked to the development of resistance [17]. Third-generation EGFR inhibitors like osimertinib were specifically designed to selectively target EGFR-sensitizing mutations, like T790M, and are currently used as a first-line treatment for EGFR-mutated NSCLC [18]. Extensive efforts have attempted to genomically characterize tumors with acquired resistance to treatment. However, most studies have demonstrated that on- and off-target genomic alterations (e.g. *PIK3CA* or *RAS* mutations, *c-Met* amplification, etc.) can explain resistance in only approximately half of patients [6]. This suggests that genomic-independent mechanisms drive the dynamic adaptations of clones within the tumor microecology to overcome treatment-associated selective pressure.

Given the intense interest to characterize these multifaceted mechanisms of resistance to anti-EGFR treatment in lung cancer, we used MCPs established from a complex, heterogeneous NSCLC model and assessed how signal dynamics affects response to anti-EGFR treatment across coexisting subpopulations of malignant cells within the tumor microecology. Our data suggest that this approach can provide mechanistic insights on how heterogeneous signal dynamics modulate responses to treatment within a tumor and offers new opportunities for identifying less responsive cancer cell subpopulations and testing combination treatments that may help overcome resistance to targeted agents.

## Methods

### Cell culture, single cell cloning, expansion procedure, and morphological determination

The NCI-H1975 non-small cell lung cancer cell line (no. CRL-5908) as well as 14 control lines, namely A547, A549, Calu, H1373, H1299, H1734, H358, H2122, H2228, H23, H522, SK-LU, H1838, and H820 were acquired from American Type Culture Collection (ATCC, Manassas, VA) and grown following manufacturer’s recommendations. Cells were cultured in a 75 cm^2^ flask in a humidified atmosphere with 5% CO_2_ at 37 °C in RPMI-1640 media (ATCC, Manassas, VA) supplemented with 10% fetal bovine serum (R&D systems, Flowery Branch, GA).

**Fig. 1:**
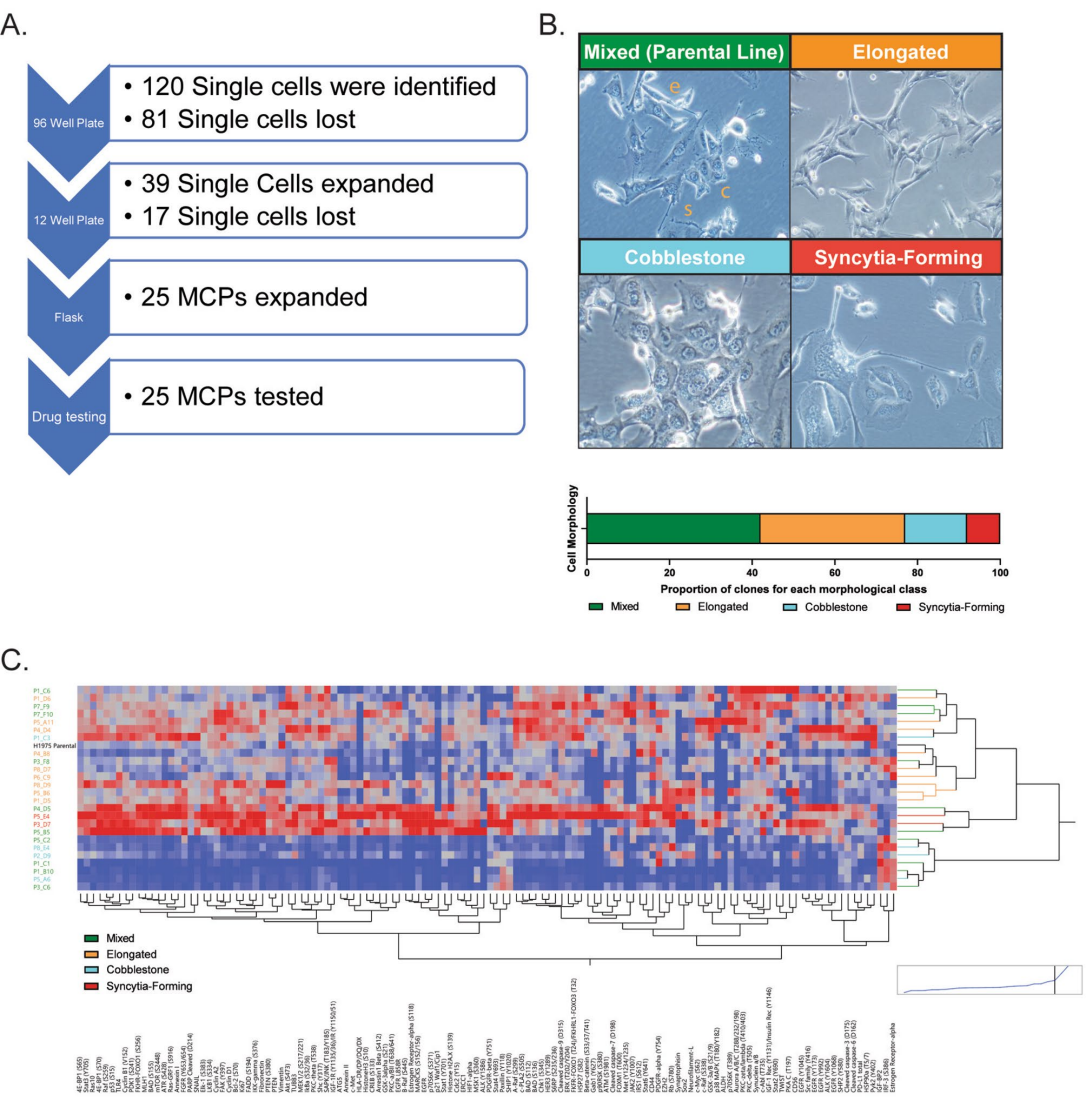
Establishment of MCPs from the H1975 commercially available NSCLC cell line. Workflow describing the different steps of the single cell cloning and expansion process with the number of individual cells or MCPs detected at each stage (Panel **A**). Brightfield images showing cellular morphology in selected clones with distinct morphological features. The parental line is shown as a reference and coexisting morphological, namely elongated (e), cobblestone (c), and syncytia-forming cells (s) are highlighted in the parental line (Panel **B**). Unsupervised hierarchical clustering analysis capturing expression or activation levels of 125 signaling molecules across MCPs. All measured proteins (x-axis) and the 25 MCPs color-coded based on their morphological characteristics (y-axis) are listed (Panel **C**)

To establish MCPs, once 75% confluent, H1975 cells were detached with 0.25% trypsin/EDTA (ATCC, Manassas, VA), centrifuged, and resuspended in complete cell media. Cell number was determined using a hemocytometer after cells were stained Trypan Blue 0.4% (Mediatech, Inc., Manassas, VA) as previously described [19]. A total of eight 96-well plates were seeded at a concentration of 1 cell per 100 µl of complete media, which was determined to be the optimal concentration for isolating single cells from a cell suspension through serial titrations experiments. Twenty-four hours after seeding, plates were independently inspected by two scientists (CB and KA) to identify wells that contained an individual cell. Wells that were confirmed to contain one cell were photographed and monitored every two days. When cells reached 50% confluency, MCPs were subject to the expansion process and were moved from 96-well plates to 12-well plates. Cells were then sub-cultured using trypsin/EDTA (ATCC, Manassas, VA, USA) at a ratio ranging between 1:4 and 1:10 based on the individual MCPs proliferation rates to 25 cm^2^ flasks, and ultimately 75 cm^2^ flasks. A summary of the process and successful establishment of MCPs is provided in Fig. 1A.

Once expanded to 75 cm^2^ flasks, MCPs were inspected and photographed using a brightfield microscope and cells were independently classified based on their morphological characteristics by two scientists (Fig S1). MCPs were classified as: cobblestone [20], syncytia-forming [21], elongated/spindle, and mixed. MCPs with 60% cells presenting with homogenous features were attributed to a single category. In the remaining MCPs, all three morphological features could be identified and were subsequently classified as mixed morphology.

### Spheroid formation and morphological analysis

Cells were seeded in low-attachment 96-well plates (ThermoFisher, Rochester, NY). Cultured cells were plated in technical replicates at a concentration of 1000 cells per well in 200 µL of media. Plates were placed in the incubator at 37 ºC with 5% CO_2_ and monitored for 48 h. After 48 h, spheroids were photographed and the media in each well was replaced with 200 µL of fresh media. On day 5, spheroids were photographed once again, and their dimension and shape were assessed using the ImageJ software version 1.54 (ImageJ.NIH.gov). Diameter, perimeter, and area were recorded for each MCP and averaged across the replicates (*n* = 2) (Fig S2). Circularity score of each MCP was then calculated as (4π × [Area]/[Perimeter]^2^), as previously described [22].

### DNA extraction and sequencing

Genomic DNA was extracted from frozen vials for 22 of the 25 MCPs, the parental H1975 cell line, and the A549 cell line that was used as a negative control using the commercially available kit QIAamp Fast DNA Tissue Kit (Qiagen, Valencia, CA, USA) following the manufacturer’s instructions. EGFR exons 20 and 21 were amplified using polymerase chain reaction (PCR) with the PCR SuperMix (Invitrogen Life Technologies, Carlsbad, CA, USA). The primer sequences are listed in Table S1. PCR was performed using a GeneAmp PCR System 9700 machine (Applied Biosystems, Waltham, MA) and the thermal cycle was programmed for 2 min at 94 °C for initial denaturation, followed by 35 cycles of 15 s at 94 °C for denaturation, 30 s at 55 °C as annealing, 1 min at 72 °C for extension, and final extension at 72 °C for 10 min. PCR products were subsequently visualized by gel electrophoresis at 150 V for 30 min on a 1% (w/v) agarose gel in 1X TAE buffer. PCR products were purified with Ampure magnetic beads (Beckman Coulter, Brea, CA) and were prepared for sequencing using BigDye Terminator v3.1 (Applied Biosystems, Waltham, MA) and forward and reverse primers. Sequencing fragments were purified by Sephadex G-50 M DNA Grade (Cytiva, Marlborough, MA) and detected by capillary electrophoresis on an ABI 3130XL Genetic Analyzer (Applied Biosystems, Waltham, MA) using Sequencing Analysis v5.4 software (Applied Biosystems, Waltham, MA). Electropherograms were analyzed for the detection of mutations using Sequencher v5.0.1 Software (Gene Codes Corporation, Ann Arbor, MI).

### Cell viability studies

Cell viability was performed using CellTiter-Glo Luminescent Cell Viability Assay (Promega, Madison, WI, USA) following manufacturer’s instructions to establish the IC50 values of osimertinib in the H1975 parental line. In brief, cell suspension was seeded in 96-well plates and plates were kept in the incubator for 24 h before treatment so that they could reach 80% confluency. Osimertinib was dissolved in dimethylsulfoxide (DMSO, ATCC, Manassas, VA), and cells were treated in a 2-fold serial dilution curve ranging from 0.002 µM to 1 µM for 72 h (Fig S3). Matched DMSO control data were collected for each dilution point and independent biological replicates (*n* = 4) were collected for each data point. Cell viability assay experiments were performed in technical replicates (*n* = 2). The same protocol was also used when MCPs were treated with the Met inhibitor tepotinib (0.02-5 µM), the IGF-1R inhibitor linsitinib (0.7–50 µM) and the pan-PKC inhibitor sotrastaurin (0.7–50 µM). After 72 h incubation with the compounds, plates were brought to room temperature for 30 min. Media was replaced with a 1:1 solution of CellTiter-Glo and fresh media, and cells were lysed on an orbital shaker at room temperature for 5 min. Luminescence signal was measured using a Beckman Coulter DTX 880 microplate reader (Beckman Coulter, Brea, CA, USA) [23]. IC50 values were calculated using a non-linear regression, four parameters curve fit method after normalization on the vehicle controls using GraphPad v9.5.1.

MCPs were treated with osimertinib at the IC50 value of the parental line (700 nM) along with the H1975 line from which they were established as control. In brief, for each MCP, a cell suspension was seeded in 96-well plates 48 h before treatment. Number of plated cells was selected based on each MCP’s proliferation rate to ensure that cells were 80% confluent before treatment. After 48 h, media was removed and replaced with either 200 µl of fresh media, 200 µl of media with 700nM osimertinib, or 200 µl of media with DMSO (< 0.01%), where the solvent amount matched the one added to the treated cells. Technical replicates (*n* = 4) were collected for each experimental condition. Cell viability after 72 h of treatment was assessed using the CellTiter-Glo protocol described above. For each cell line, a Drug Sensitivity Score was calculated to capture differences in response between MCPs and the parental line from which they were established. Drug sensitivity scores were calculated in a two-step process for each MCP: cell viability rates were first normalized to the matched vehicle control-treated samples and subsequently to the values obtained from the parental line. This process allowed us to report cell viability for each MCP as a percent change of the parental line.

### Cell lysate preparation, protein extraction, and reverse phase protein microarray (RPPA)

MCPs, H1975, and 14 controls were seeded in technical replicates (*n* = 3) in 6-well plates and cultured until 80% confluent. Cells were washed twice with Dulbecco’s phosphate-buffered saline (PBS) (Invitrogen Life Technologies, Carlsbad, CA, USA) and lysed in a 1:1 solution of Tissue Protein Extraction Reagent (T-PER) (Thermo Fisher Scientific, Waltham, MA, USA) and in 2X Tris-Glycine SDS Sample buffer (Invitrogen Life Technologies, Carlsbad, CA, USA) supplemented with 5% β-mercaptoethanol (Sigma-Aldrich, St. Louis, MO, USA). Cell lysates were then boiled for 8 min at 100 °C. MCPs and control cells were immobilized onto nitrocellulose glass slides along with a bovine serum albumin (BSA) reference standard (range 1–0.125 mg/mL) to assess the amount of protein in each sample. Samples were subsequently brought to a final concentration of 500 µg/mL using the same lysis buffer and stored at − 80 °C until further processed.

Reverse Phase Protein Microarray (RPPA) analysis was performed to capture expression and activation levels of 125 signaling molecules across the MCPs, parental cell, and 14 NSCLC controls. Cell lysates were immobilized in technical replicates (*n* = 3) onto nitrocellulose-coated glass slides (Oncyte Avid, Grace Bio-Labs, Bend, OR, USA) using an automated system (Aushon 2470 arrayer, Quanterix, Billerica, MA, USA). Selected arrays were probed with Sypro Ruby Protein Blot Stain (Molecular Probes, Eugene, OR, USA) following the manufacturer’s directions to quantify the protein amount of each sample and used for normalization purposes. Remaining arrays were probed with one polyclonal or monoclonal primary antibody targeting a protein of interest using an automated Epredia Autostainer 360 system (DA Breda, Netherlands). Arrays were first treated with Reblot Antibody Stripping solution (Invitrogen Life Technologies, Carlsbad, CA, USA) for 15 min at room temperature, followed by two washes with PBS and incubated for at least 4 h in I-Block (Tropix, Bedford, MA). Using the automated staining system, arrays were then treated with 3% hydrogen peroxide (Sigma-Aldrich, St. Louis, MO), a biotin blocking system (Dako Cytomation, Carpinteria, CA), and an additional serum-free protein block (Dako Cytomation, Carpinteria, CA). Finally, arrays were probed with 125 antibodies (Table S2), validated for their specificity against the target protein by Western blotting, as previously described [24]. Biotinylated anti-rabbit, anti-mouse, or anti-rat secondary antibody (Vector Laboratories, Inc. Burlingame, CA) coupled with a commercially available tyramide-based avidin/biotin amplification system were then used to quantify signaling molecules within each sample. Specifically, arrays were incubated with the Vectastain Elite ABC peroxidase system prepared at a 1:50 dilution in PBS for 30 min (Vector Laboratories, Newark, CA) followed by 10 min incubation with a tyramide signal amplification kit (TSA™ Plus Biotin, Akoya Biosciences, Marlborough, MA) resuspended in 300 µl of DMSO (ATCC, Manassas, VA) and diluted 1:200 in dilution buffer (Akoya Biosciences, Marlborough, MA). Signal detection was obtained using IRDye 680RD Streptavidin (LI-COR Biosciences, Lincoln, NE) prepared according to the manufacturer’s recommendation. Antibody and Sypro Ruby stained slides were scanned on a Tecan laser scanner (TECAN, Mönnedorf, Switzerland). Images were analyzed as previously described [25].

### Statistical analysis

Unsupervised hierarchical clustering analysis using the Ward method, Spearman rank-order correlation coefficients of RPPA-based continuous data, and partition tree analysis were performed in JMP v17 (SAS Institute Inc., Cary, NC, USA). The two-tailed Kruskal Wallis rank test, a non-parametric one-way analysis of variance method, was performed in SPSS v28 to identify changes in protein expression/activation across MCPs based on their morphological characteristics and drug sensitivity score. Based on response to treatment, MCPs were classified in four groups as follows: super-sensitive (drug sensitivity score < 0.5), sensitive (drug sensitivity score between 0.5 and 0.8), neutral (drug sensitivity score between 0.8 and 1.2), and resistant (drug sensitivity score > 1.2) lines. To capture molecular events associated with different degrees of susceptibility to treatment, a three-group comparison using the Kruskal Wallis rank test was performed between super sensitive, sensitive, and resistant cells. Alpha levels for significance were set at 0.05. RPPA values were displayed using bar graphs created in GraphPad version v.10; mean and standard error of the mean (SEM) are shown along with Bonferroni adjusted p-values to account for multiple comparisons. Unrooted phylogenetic neighbor joining trees were built in R v4.3.2 using the nj function of the ape package (v 5.7-1, Paradis & Schliep 2019) based on Euclidean distances. NJ tree was first built using 115 RPPA endpoints (all RPPA for which the parental line did not have a 0 value) and then using the 33 proteins including receptor tyrosine kinases (RTKs) and downstream substrates only. Data were first normalized for each RPPA by dividing each MCP value by the parental line, then using the scale function in R across the entire dataset used for each analysis.

**Fig. 2:**
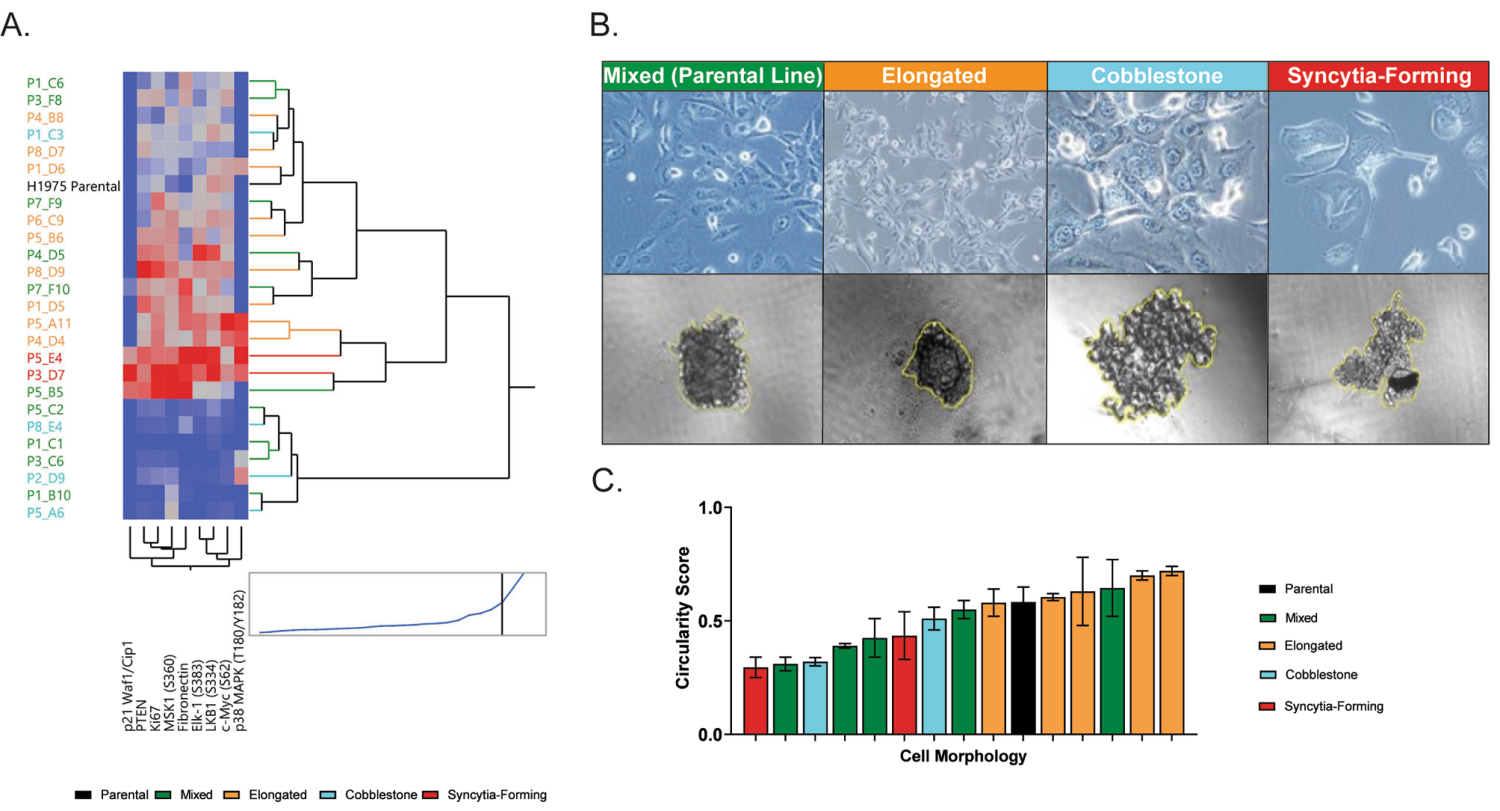
Phenotypic and morphological characteristics of the 25 MCPs established from the H1975 cell line. Unsupervised clustering analysis capturing expression or activation levels of 10 signaling molecules that reached statistical significance (*p* < 0.05) when MCPs with different morphological characteristics were compared (Panel **A**). Examples of 3D structure formed by MCPs grown in low attachment plates along with their morphological characteristics (Panel **B**) and circularity score (Panel **C**)

**Fig. 3:**
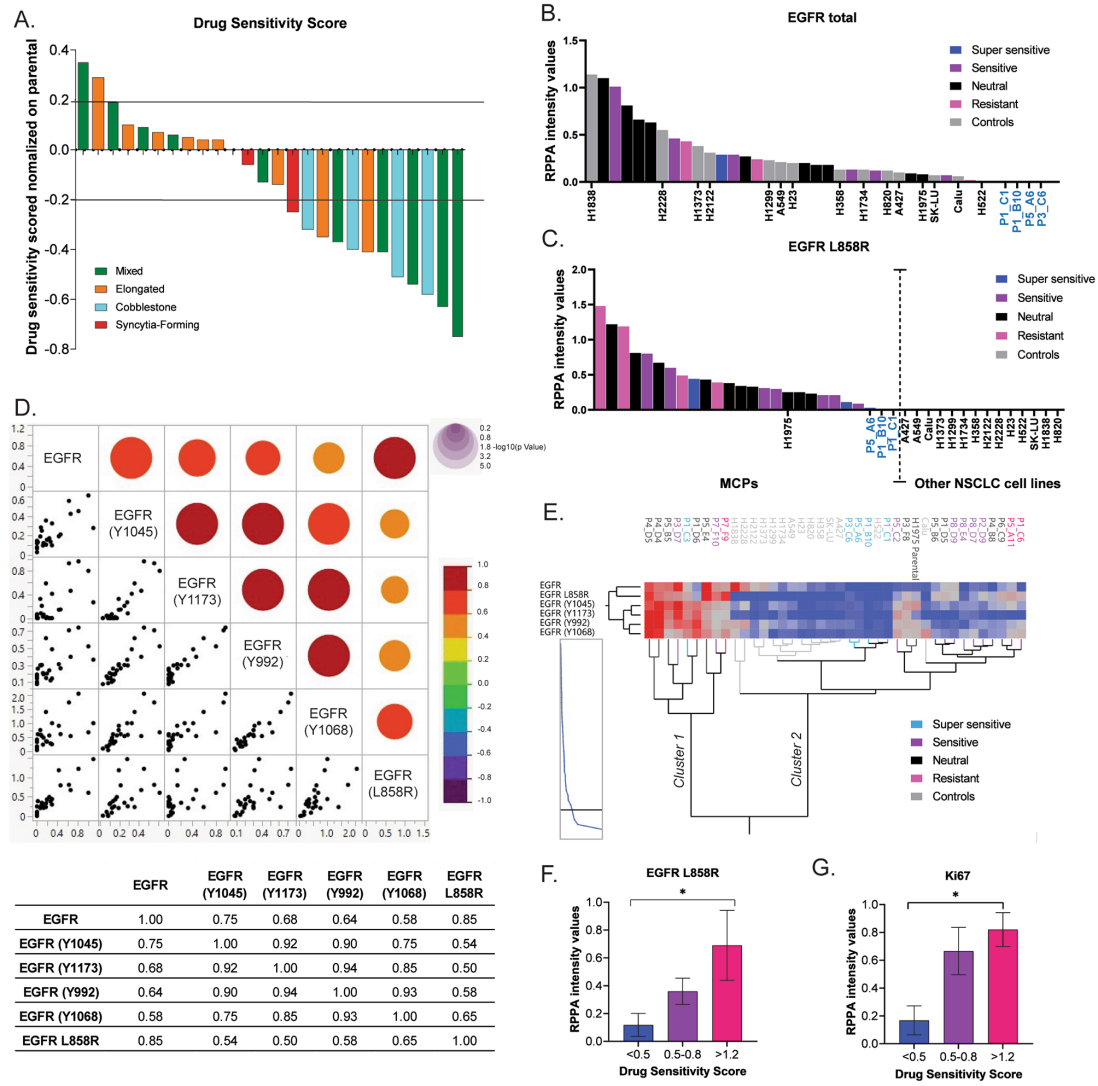
Response of MCPs to the anti-EGFR compound osimertinib compared to the parental line from which they were established. Waterfall plot illustrating the percentage change in cell viability in the MCPs after 72 h of treatment with osimertinib; values for MCPs are normalized to matched vehicle control-treated samples and subsequently to post-treatment cell viability values of the parental line (Panel **A**). Dynamic range of expression of the EGFR receptor (Panel **B**) and its L858R mutant variant (Panel **C**) by RPPA intensity values (y-axis) across the 25 MCPs and 14 NSCLC control cell lines (x-axis), which are delineated with a dotted vertical line to signify low vs. high expression of EGFR. Correlation matrix and correlation coefficients (ρ) capturing levels of association between expression and activation of the EGFR receptor. Red dots indicate positive correlations; the dimension of the dots is proportional to the strength of the association (Panel **D**). Unsupervised hierarchical clustering analysis displaying changes in the EGFR receptor expression and activation across 25 MCPs and 14 NSCLC control cell lines (Panel **E**). Bar graph with mean and standard error of the mean (SEM) of expression levels of EGFR L858R in MCPs based on their levels of sensitivity to osimertinib (Panel **F**). Bar graph with mean and SEM capturing expression levels of Ki67 in MCPs with different levels of sensitivity to treatment (Panel **G**)

## Results

### Monoclonal cell populations establishment and characterization

As a model system for tracing and dissecting coexisting mechanisms of resistance to anti-EGFR treatment, we established monoclonal cell subpopulations using single cell cloning and expansion techniques from the commercially available H1975 NSCLC cell line. The H1975 cell line was selected as a model system for this analysis as it harbors two key alterations of the *EGFR* gene associated with response to anti-EGFR treatment: L858R and T790M. The L858R oncogenic point mutation on exon 21 affects the region in close proximity to the activation loop of the kinase domain of the receptor [26]. T790M, the gatekeeper mutation, is found on exon 20 and is known to be associated with acquired resistance to first and second-generation EGFR inhibitors through steric hindrance to the inhibitor binding site [27]. The H1975 cell line lacks previously identified off-target genetic alterations associated with resistance to osimertinib [6, 28] (e.g. *KRAS*, *BRAF*, and *PIK3CA* mutations, *c-Met* amplification, previously reported fusions, etc.) according to data collected on the COSMIC database [29]. After successfully seeding 120 individual cells from the H1975 cell line, 25 were subsequently expanded and grown as MCPs (Fig. 1A and Fig S1). When EGFR mutational status was compared between the parental line and 22 MCPs (Table S1), the L858R and T790M mutations were detected across all tested samples, as expected. However, C797S, a known secondary on-target mutation associated with a lack of response to osimertinib [17], was not detected in any of the MCPs. Neither mutation was detected in the A549 cell line used as a negative control (Fig S4A). This result emphasizes that all MCPs share lineage through a uniquely identifiable genomic fingerprint in the H1975 cell line that is distinctly absent in the 14 other NSCLC cell lines that we evaluated. To assess similarities between the H1975 parental cells and the MCPs, we next compared the signaling profile of the MCPs against 14 control cell lines using Reverse Phase Protein Array (RPPA). Unsupervised hierarchical clustering analysis of 125 signaling molecules indicated that the MCPs and parental line were contained within the same cluster and had distinct molecular characteristics compared to control cell lines, which had a diverse genomic background. This confirmed the common origin of the MCPs from the parental line (Fig S4B).

### Monoclonal cell populations have unique morphological characteristics

We next used brightfield microscopy to assess the variation in the morphological characteristics of the MCPs compared to that of the parental line. The parental line contained cells with three distinct morphological characteristics including elongated/spindle, cobblestone, and syncytia-forming cells [20, 21] (Fig. 1B). Ten MCPs mimicked the morphological characteristics of the parental line whereby cells with all three morphological types were identified (Fig S5). The remaining 15 MCPs were characterized by a more homogenous cellular morphology: elongated/spindle cells (*n* = 9 MCPs), cobblestone cells (*n* = 4), or syncytia-forming cells (*n* = 2) (Fig. 1B and Fig S5).

To investigate potential relationships between morphological characteristics and function, we used RPPA to measure the expression and activation levels of 125 cancer-associated proteins across the 25 MCPs and the parental line (Table S2). Unsupervised hierarchal clustering analysis showed heterogeneous signaling architectures across the 25 MCPs with mixed morphology spread across the three main clusters (Fig. 1C). MCPs with elongated characteristics were contained within the first cluster and presented with heterogeneous activation of signaling molecules. Syncytia-forming cells were contained within the second cluster and had overall high signaling activities. Lastly, MCPs with predominant cobblestone morphology were mostly contained within the third cluster and were characterized by low overall activation of the signaling molecules analyzed (Fig. 1C). Kruskal–Wallis one-way analysis of variance was then used to compare the expression and activation of signaling molecules across the four morphological groups. Of the 125 proteins measured by RPPA, 10 were statistically significant (*p* < 0.05) and increased signaling activity, especially of mitogenic and stress-activated protein kinases (e.g. p38 MAPK (T180/Y182), Elk-1 (S383), c-Myc (S62), MSK1 (S360), in MCPs belonging to the syncytia-forming group compared to cells with cobblestone morphology (Fig. 2A).

We next assessed the ability of 14 MCPs and the parental line to form 3D structures when grown in low-attachment plates. All MCPs tested successfully generated spheroids within five days from when cells were seeded (Fig. 2B and Fig S2). To quantify structural differences in spheroids derived from different MCPs, we calculated a circularity score where spheroids with a score of 1 were classified as perfectly circular. MCPs with elongated morphology generated spheroids with a more circular structure compared to MCPs derived from cells with other morphological characteristics (*p* = 0.05) (Fig. 2C). Taken together, our data suggest that MCPs derived from the same parental cell line have unique morphological characteristics, and these features may be driven by the activation of specific signaling molecules like mitogenic and stress-activated protein kinases.

### Monoclonal cell populations established from the H1975 NSCLC cell line have distinct sensitivity to anti-EGFR treatment

Given that the H1975 cell line harbors the T790M mutation known to be associated with response to the EGFR inhibitor osimertinib and that understanding mechanisms of resistance to this targeted agent remains a priority in the clinic, we next assessed sensitivity to osimertinib across the 25 MCPs compared to that of the parental line. MCPs were treated alongside the parental line with 700 nM osimertinib, corresponding to the IC50 value of the H1975 after normalization to the matched DMSO control (Fig S3). Cell viability at 72 h was then compared between MCPs and the parental line using a drug sensitivity score. In brief, for each MCP, cell viability in response to osimertinib was first normalized to matched vehicle control-treated samples and subsequently to post-treatment cell viability values of the parental line. As shown in Fig. 3A, response to treatment across MCPs revealed substantial differences in drug sensitivity compared to the parental line. Of interest, ten of the 25 (40%) MCPs had a drug sensitivity score that was similar to the parental cells from which they were established (< 20% change), while for three MCPs (12%), the drug sensitivity score was ≥ 20% compared to the parental line (Fig. 3A and Table S3). The remaining MCPs were considered more sensitive to treatment with osimertinib than the parental line and cell viability decreased by 40% in 8 MCPs (32%) and by 63% and 75% in the two most sensitive subpopulations (Fig. 3A). While drug sensitivity scores were heterogenous in cells with mixed morphology, MCPs with cobblestone features were generally more sensitive to treatment compared to MCPs with an elongated morphology. Taken together, this data suggests that MCPs established from the same tumor have different degrees of susceptibility to osimertinib and may be used to gather insights on coexisting mechanisms of response to treatment within the same tumor.

### Expression and activation of EGFR is highly heterogeneous across monoclonal cell populations

Since osimertinib is designed to modulate EGFR activity, we next used the RPPA data to evaluate changes in EGFR expression across MCPs, the parental line, and the 14 NSCLC control lines with diverse genomic background. EGFR expression was measured using two commercially available antibodies. The first antibody was designed to recognize an unmodified epitope on the EGFR molecule unrelated to the mutation site (referred to as EGFR), while the second antibody was designed to specifically recognize the L858R mutant form of the receptor (referred to as EGFR L858R). Unmodified and mutant EGFR levels were very heterogeneous across models, and the expression of EGFR quantified by RPPA in the MCPs had a dynamic range spanning from 0.25 to almost 14 times that of the parental line (Fig. 3B). As expected, the EGFR-amplified H1838 NSCLC control cell line had the highest expression levels of EGFR compared to the other models. Notably, however, a number of MCPs had EGFR levels that were similar to those of the H1838 cells despite H1975 having a non-amplified EGFR status. Furthermore, EGFR L858R was not detected in the control cell lines but was apparent in the H1975 cell line (Fig. 3C), which was the only model in our NSCLC panel harboring the L858R mutation [30]. Of interest, total EGFR and EGFR L858R mutant form was not detected in two MCPs, namely P1C1 and P1B10, both of which were more sensitive to treatment with osimertinib than the parental line.

We next calculated Spearman Rho correlation coefficients (ρ) using the RPPA data to assess levels of association between expression and activation of EGFR. Activation of EGFR was determined through RPPA measurement of four phosphorylated tyrosine residues (Y992, Y1045, Y1068, and Y1173) associated with EGFR stimulation/autophosphorylation and downstream signaling events [31–34]. As expected, EGFR L858R levels were highly correlated with overall EGFR expression in the MCPs (ρ = 0.85), and high correlation levels were also detected between phosphorylation sites of the receptor (ρ between 0.74 and 0.94) (Fig. 3D). However, only modest associations were detected between EGFR L858R levels and receptor activity (ρ between 0.51 and 0.65).

Considering the different responses to EGFR inhibition across MCPs, which may suggest variations in EGFR expression and activity, we next sought to compare EGFR expression and activation levels across MCPs and assessed their effect in response to osimertinib. To correlate drug response of the MCPs with their signaling architecture, we subclassified MCPs based on their sensitivity compared to the parental line (cell viability after treatment in MCPs/cell viability after treatment in parental cells) into four groups: super-sensitive (drug sensitivity score < 0.5), sensitive (drug sensitivity score between 0.5 and 0.8), neutral (drug sensitivity score between 0.8 and 1.2), and resistant (drug sensitivity score > 1.2) (Table S3). Although arbitrary, we believe these four groups capture different levels of sensitivity to osimertinib across MCPs.

We then used unsupervised hierarchical clustering analysis to capture the expression and activation of EGFR across MCPs and control lines. MCPs and control lines were clustered into two main groups with most of the super-sensitive MCPs (80%) clustering with the control cell lines and presenting with overall low levels of expression and activation of the receptor compared to expression and activation of the other MCPs and the parental line (Fig. 3E). A subgroup of MCPs (Fig. 3E, Cluster 1) had high expression and activation of the receptor compared to the remaining MCPs and control samples. The remaining MCPs presented with various degrees of EGFR expression and activation. For example, the cluster including the parental cells and MCPs P3F8 and P5C2 was characterized by low EGFR expression but relatively high levels of activation. Lastly, we used Kruskal–Wallis one-way analysis of variance to compare expression and activation levels of EGFR in super-sensitive, sensitive, and resistant MCPs. EGFR L858R expression emerged as the only analyte that was statistically different between response groups where super-sensitive MCPs had significantly lower levels of EGFR L858R compared to the resistant groups (*p* = 0.045) (Fig. 3F). Along with increased expression of EGFR L858R, MCPs that were less sensitive to treatment with osimertinib had higher proliferation rates, measured as expression of Ki67, compared to the more sensitive MCPs (*p* = 0.023) (Fig. 3G). Taken together, this data suggests that even within the same tumor, anti-EGFR treatment may be more effective in clones with lower proliferation rates and lower expression of the mutant form of the EGFR. Mapping expression levels of EGFR L858R across clones within the same tumor along with their levels of proliferation may provide important insights for predicting and monitoring response to anti-EGFR treatment in NSCLC patients.

### Monoclonal cell populations that are sensitive to osimertinib have low activation of EGFR dimerizing partners

**Fig. 4:**
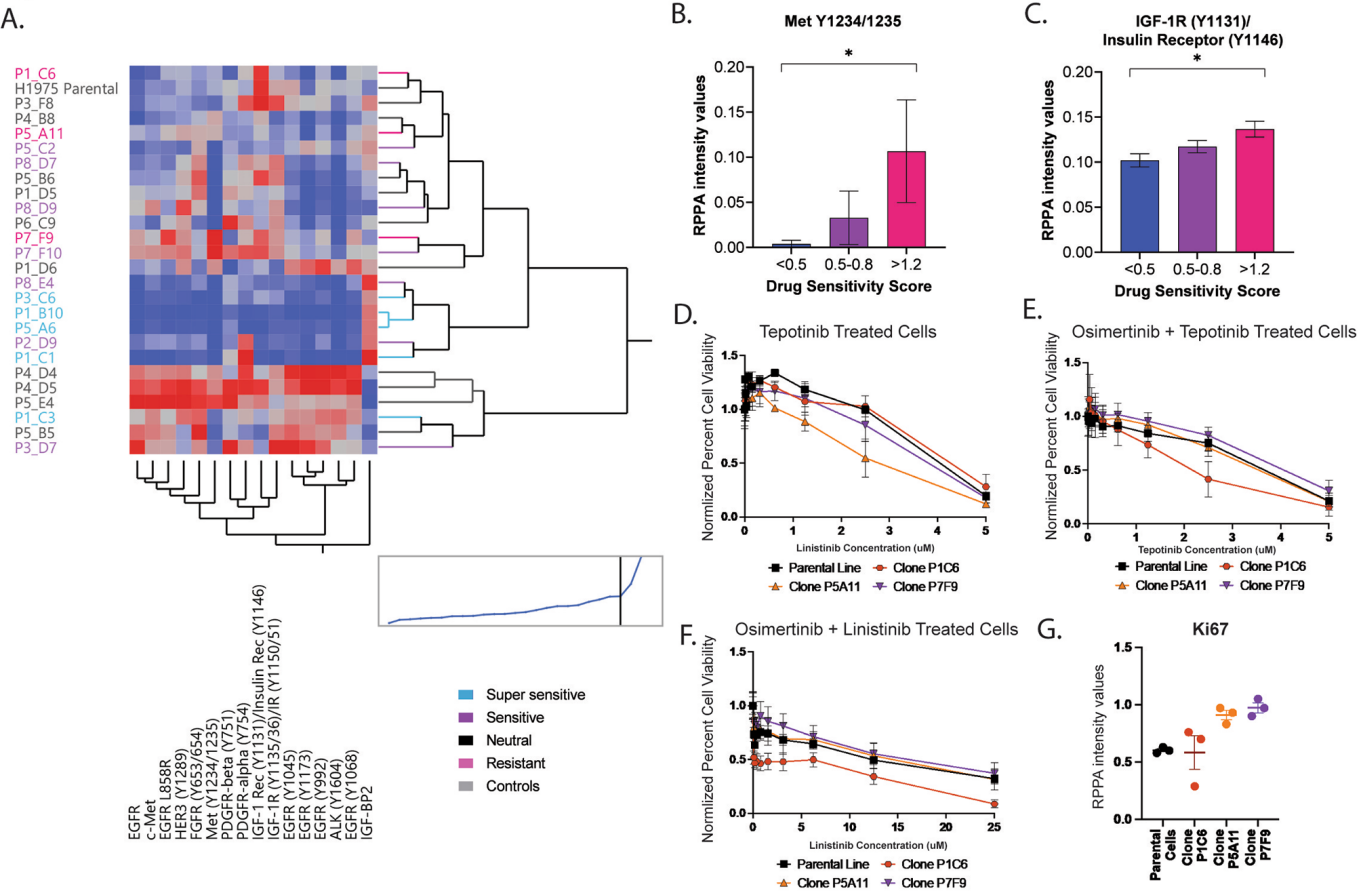
Activation levels of RTKs across MCPs with different levels of susceptibility to osimertinib. Unsupervised hierarchical clustering analysis capturing activation levels of RTKs in MCPs and parental line. MCPs are color-coded based on their levels of response to osimertinib on the x-axis; measured analytes are listed on the y-axis (Panel **A**). Bar graphs with mean and SEM capturing levels of phosphorylated of Met and IGF-1R/insulin receptor in clones with different levels of sensitivity to osimertinib (Panel **B** and **C**, respectively; *p* = 0.03 for both comparisons). Dose-response plots capturing response in selected clones treated to the Met inhibitor tepotinib as single agent (Panel **D**) and in combination with osimertinib (Panel **E**) and to the IGF-1R inhibitor linsitinib in combination with osimertinib (Panel **F**). Bar-graphs with mean and SEM capturing Ki67 levels across MCPs treated with the Met and IGF-1R inhibitors (Panel **G**)

As the correlation between high EGFR L858R levels and reduced response to osimertinib was unexpected, we next assessed whether high levels of mutant EGFR were associated with activation through heterodimerization of other receptor tyrosine kinases (RTKs), which is a known mechanism of resistance to anti-EGFR treatment in lung cancer [35]. Using the continuous RPPA data, we assessed activation levels of eight RTKs, including c-Met (Y1234/1255), FGFR, HER3 (Y1289), IGF-1R/Insulin receptor (Y1131/Y1146 and Y1135/1136-Y1150/1151), PDGFR-alpha (Y754), PDGFR-beta (Y751), and ALK (Y1604) along with EGFR expression and activation using unsupervised hierarchical clustering analysis. MCPs with high expression levels of EGFR and its mutant form had increased activation across all receptors, suggesting global RTK activity in these samples (Fig. 4A). MCPs that were classified as resistant were contained in different clusters and overall were characterized by increased activation of c-Met (Y1234/1255) and IGF-1R/IR (Y1131/Y1146). On the contrary, MCPs that were super-sensitive to osimertinib were contained within the same cluster, with the exception of P1C3, and demonstrated low signaling activity of the different RTKs. Of interest, MCPs with lower RTK signaling had higher expression levels of IGF-BP2, a regulator of IGF-1R activity [36]. Kruskal–Wallis one-way analysis of variance confirmed that activation of c-Met (Y1234/1255) and IGF-1R/IR (Y1131/Y1146) were significantly lower in the super-sensitive MCPs compared to the resistant group (Fig. 4B and C). Of interest, while expression of c-Met was associated with EGFR and EGFR L858R expression levels across MCPs (ρ = 0.87 and 0.81, respectively), correlation between EGFR expression and phosphorylated c-Met (Y1234/1255) was much weaker (ρ = 0.5). Similarly, weak correlations were also found between EGFR expression and phosphorylation of IGF-1R/IR (Y1131/Y1146) (ρ < 0.5) (Fig S6).

We then assessed whether inhibition of hyperactivated c-Met and IGF1R/IR was sufficient to induce response to a targeted agent in cells with high overall RTK activity. We first compared the responses of the three resistant MCPs and the parental line to the c-Met inhibitor tepotinib as a single agent and in combination with 700 nM osimertinib (corresponding to the IC50 value of the parental line). To capture changes in cell viability in response to the combination treatment, output data was normalized on the cell viability obtained when cells were treated with osimertinib alone. Of interest, P5A11 was characterized by intermediate activation of c-Met, and low phosphorylation of IGF1R/IR was more sensitive than the parental line and the other MCPs to treatment with tepotinib as a single agent (Fig. 4D). When cells were treated with tepotinib in combination with osimertinib, co-administration of the two inhibitors did not increase response in P5A11 (Fig. 4E). In contrast, P1C6 presented with high phosphorylation levels of IGF-1R/IR (Y1131/Y1146) and low c-Met (Y1234/1235) activation and was the most responsive MCP when treated with osimertinib in combination with the IGF-1R selective inhibitor linsitinib (Fig. 4F). Cell viability of P7F9, characterized by high signaling activity of IGF-1R/IR and c-Met and increased proliferation rates (Fig. 4G) was not affected by the addition of either inhibitor (Fig. 4E and F). Taken together, this data suggests that coexisting subpopulations of cancer cells within the same tumor have different levels of sensitivity to targeted inhibitors even in the absence of genomic alterations (e.g., *c-Met* amplification) known to drive resistance to anti-EGFR treatment. Understanding the role and aggressiveness (e.g., proliferation rates) of these different cell subpopulations may hold important insights for selecting and timing combination treatments targeting the most aggressive cells in the clinic.

### EGFR downstream signaling molecules are differentially activated in monoclonal cell populations with diverse levels of sensitivity to osimertinib

**Fig. 5:**
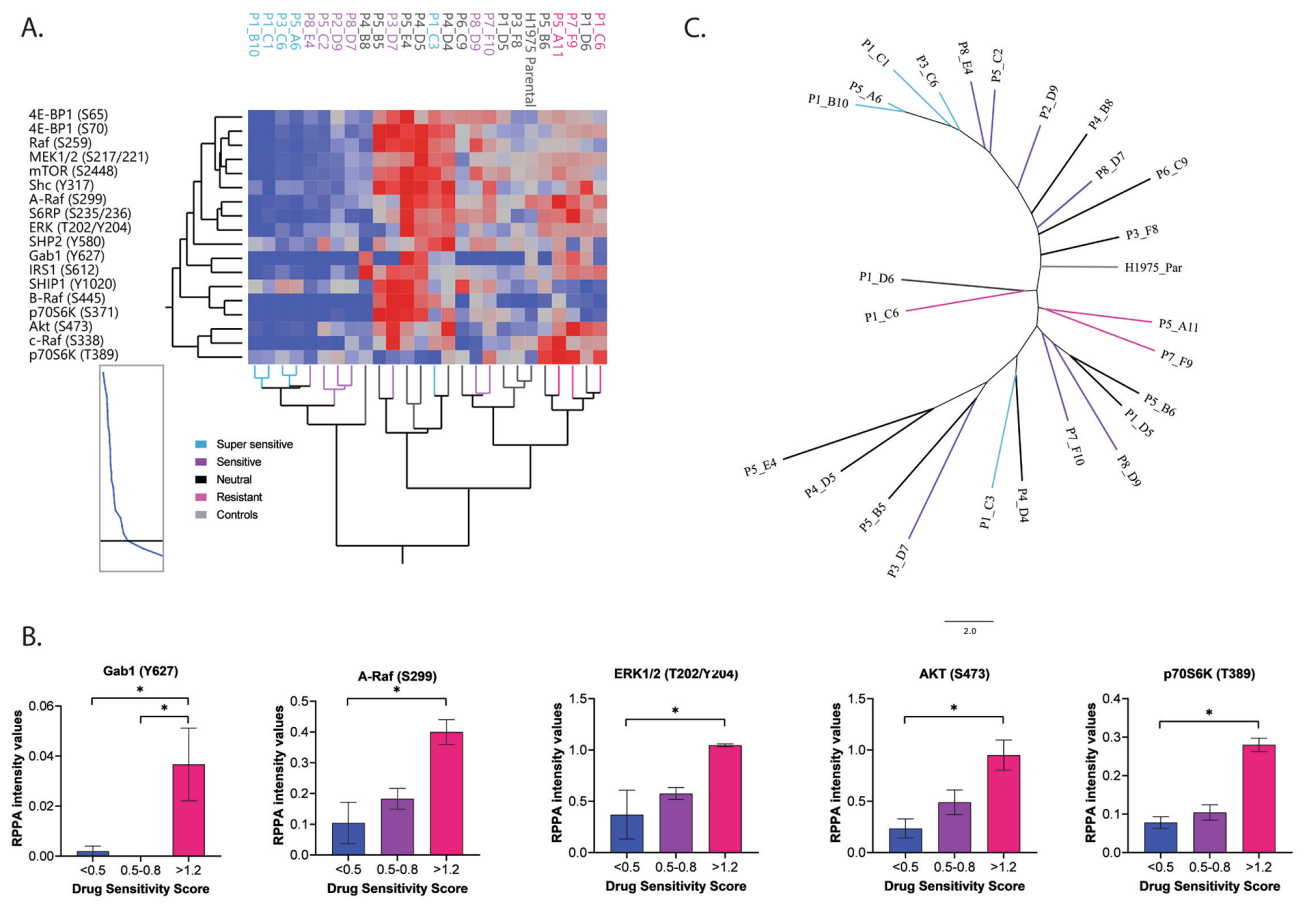
Signaling dynamics of the MAPK and AKT pathway across MCPs with different susceptibility to osimertinib. Unsupervised hierarchical clustering analysis capturing activation levels of 17 signaling proteins known to be downstream substrates of EGFR with MCPs color-coded based on their osimertinib response (Panel **A**). Bar graphs with mean and SEM for proteins whose activation or expression reached statistical significance (*p* < 0.05) after post hoc analysis across MCPs with different levels of sensitivity to osimertinib (Panel **B**). Unrooted phylogenetic neighbor joining tree depicting relatedness across models including MCPs and the parental line. MCPs were color-coded based on their levels of response to treatment with osimertinib (Panel **C**)

Given that EGFR activation and RTK activity in general could not fully differentiate resistant group from the remaining MCPs, we next used the RPPA output data to assess whether activation of main EGFR downstream signaling pathways, namely the MAPK and AKT-mTOR axes, were associated with response to osimertinib in MCPs established from the H1975 line. As shown in Fig. 5A, MCPs classified as super-sensitive or sensitive were largely contained in the same cluster and showed overall low levels of activation of different members of the MAPK and AKT-mTOR signaling pathways, which is in line with reduced RTK activity in these cells. Resistant MCPs were all contained within the same cluster, and this cluster was largely driven by high levels of activation of c-Raf (S338) and the pro-survival signaling molecules Akt (S473) and p70S6 kinase (T389) (Fig. 5B). Of interest, when levels of activation of these signaling molecules were compared between MCPs and a panel of NSCLC control lines, the H820 cell line (the other model harboring an EGFR oncogenic alteration -exon 19 deletion- and the T790M mutation in our panel), was the only other cell line that clustered with the resistant MCPs (Fig S7). Lastly, when the activation of these signaling molecules was tested across biological replicates for selected MCPs using unsupervised clustering, matched MCPs were largely contained within the sample cluster (Fig S8). Kruskal–Wallis one-way analysis of variance with post hoc analysis confirmed that phosphorylation levels were significantly higher in the resistant MCPs compared to more sensitive lines for the adaptor molecule Gab1 (Y627) (*p* = 0.01), the MAPK signaling molecules A-Raf (S229) and Erk 1/2 (T202/Y204) (*p* = 0.03 and 0.05, respectively), as well as Akt (S473) and the mTOR substrate p70S6K (T389) (*p* = 0.03) (Fig. 5B); unadjusted p values are reported (Table S2). Taken together, this data suggests that activation of downstream signaling molecules, more than receptor activity, can identify heterogeneous responses in an individual tumor.

### Epithelial-to-mesenchymal transition is associated with lack of response to osimertinib

Considering that the activation of EGFR downstream signaling substrates were associated with response to osimertinib across MCPs, we next compared activation levels of all 125 signaling proteins across response classes. Resistant lines were again generally contained within the same cluster, confirming that signaling events outside of the drug target itself play a primary role in modulating responses to treatment (Fig S9). We then used unrooted phylogenetic neighbor joining trees to assess relatedness across all models analyzed including MCPs and the parental line (Fig. 5C). We decided to not root the trees, as the common ancestor in our dataset is unknown. A phylogenetic tree generated using 115 proteins measured by RPPA confirmed that MCPs sensitive to osimertinib were generally reported as more related (blue branches of the tree). Similar results were also obtained when the analysis was restricted to RTKs and downstream signaling molecules, suggesting robustness of the data (Fig S10). The two MCPs with the lowest response rates, namely P7F9 and P5A11, were also closely related based on their molecular profiles. Whether these two MCPs were derived from the same subpopulation within the H1975 parental line, or they were the product of distinct clones, our data suggest that MCPs with similar molecular profiles are robust model systems for mechanistically targeting subpopulations within a complex human tumor that has resistance to specific targeted compounds.

**Fig. 6:**
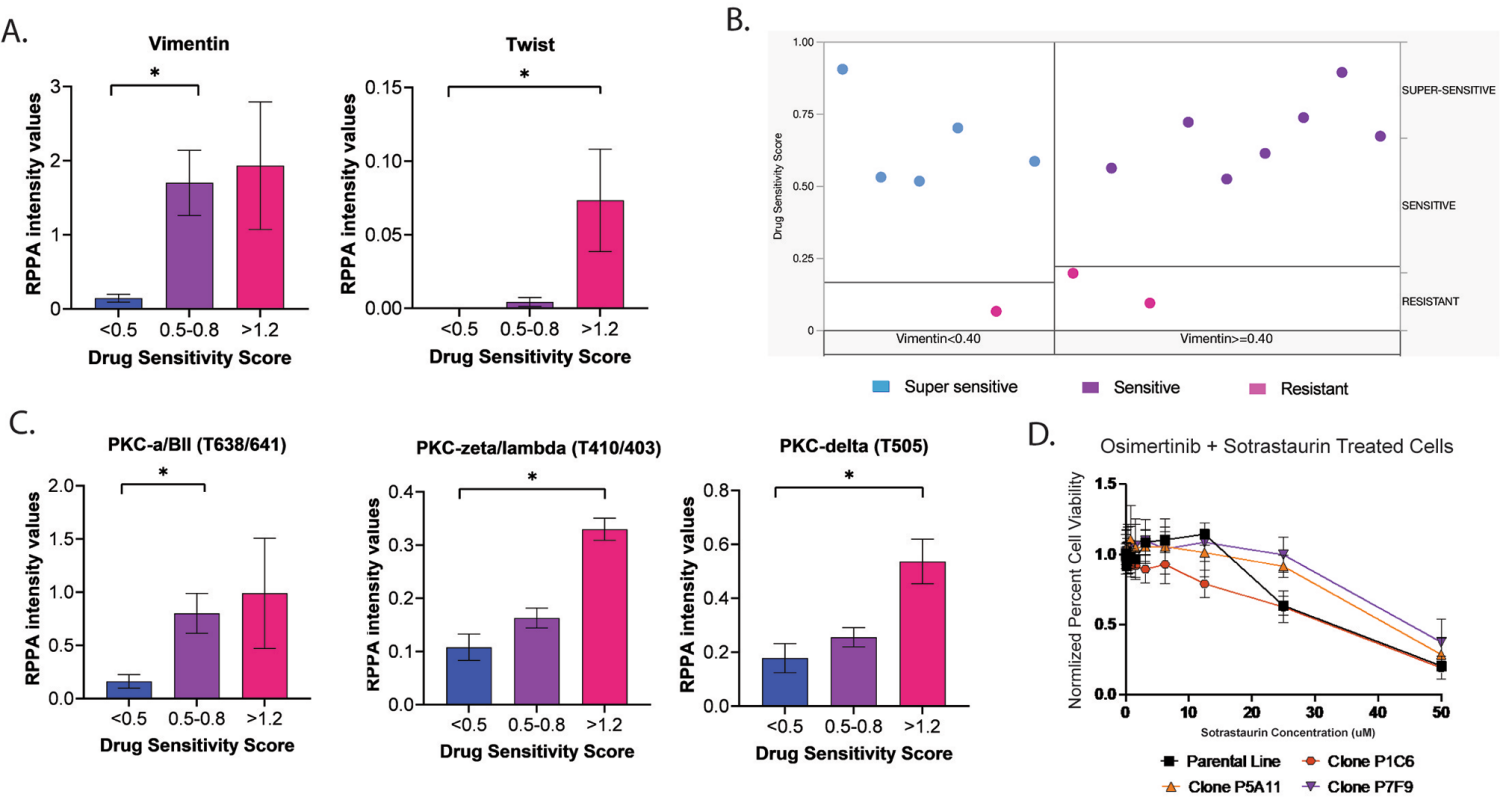
Expression and activation of proteins involved in EMT in MCPs with different levels of sensitivity to treatment with osimertinib. Bar graphs with mean and SEM for vimentin and TWIST (*p* = 0.02 and *p* = 0.01, respectively), two main EMT effectors, in MCPs with different levels of susceptibility to treatment (Panel **A**). Among the 125 proteins measured by RPPA, a partition tree analysis identified vimentin as the best predictor of response to treatment with osimertinib across MCPs belonging to different response classes (Panel **B**). Bar graphs with mean and SEM for different PKC isoforms (PKC α/β II (T638/641), *p* = 0.03; PKC ζ/λ (T410/403), *p* = 0.01; and PKC δ (T505), *p* = 0.01) (Panel **C**). Drug-response curve for selected clones treated with osimertinib in combination with the pan-PKC inhibitor sotrastaurin (Panel **D**)

When expression/activation of the remaining 88 signaling molecules was assessed, 19 were statistically significant (Table S2). Of interest, expression of proteins involved in epithelial-mesenchymal transition (EMT), including vimentin and the transcription factor TWIST, were significantly lower in MCPs that were super-sensitive to treatment compared to that of the resistant group (*p* = 0.01 and 0.02, respectively) (Fig. 6A). While PDGFR activity was highly heterogeneous across MCPs and control lines, expression of EMT markers in resistant MCPs was not necessarily associated with increased PDGFR activity (Fig S11). This lack of concordance may be attributed to the diverse functions PDGFRs have in cancer cells based on ligands they bind to, dimerization partners, and crosstalk with different signaling pathways [37–40].

When partition tree analysis was applied to the entire dataset, vimentin emerged as the best classifier for identifying super-sensitive MCPs (Fig. 6B). In addition, phosphorylation levels of Protein Kinase C (PKC) isoforms, a class of proteins previously linked to resistance to anti-RTK targeted agents [41, 42] and EMT in cancer [43, 44], were also reduced in super-sensitive MCPs (Fig. 6C). While the parental line and P1C6 showed some levels of sensitivity to the pan-PKC inhibitor sotrastaurin, targeting these signaling molecules did not effectively affect growth kinetics and survival of the two MCPs with the highest levels of resistance to osimertinib (Fig. 6D). This observation may explain some of the inconsistent results seen in the clinic with these inhibitors [45–50].

## Discussion

Using a systems-based approach, this work explored signaling dynamics and drug sensitivity between cell subpopulations originated from a commercially available, patient-derived NSCLC cell line [16]. Our work demonstrates that MCPs are a suitable model system for characterizing morphological features and levels of sensitivity to treatment across co-existing subpopulation of cells established from a complex human cancer. Using a pathway-centered approach, our work also identified molecular mechanisms, like EMT, a non-genetically driven known mechanism of resistance to anti-cancer compounds including EGFR inhibitors [51], uniquely attributable to subpopulations of cancer cells less responsive to treatment [52–54]. Taken together, our data suggest that MCPs represent a suitable model system for characterizing heterogeneous biomolecular behaviors in preclinical studies and for identifying and functionally testing biological mechanisms associated with resistance to targeted therapeutics.

In the clinic, devising personalized approaches for patients affected by *EGFR* mutant NSCLCs resistant to targeted therapy remains challenging. Many studies have characterized the genomic profile of tumors with innate and acquired resistance to anti-EGFR treatments [55–57] though emerging evidence suggests that resistance can only be explained at the genomic level (e.g. secondary EGFR mutations, *c-Met* or *ErbB2* amplification, *KRAS* or *PIK3CA* mutations, etc.) in about half of patients [58]. This suggests that genomic-independent events able to modulate the activation levels of the drug target itself and downstream signaling molecules play a primary role in shaping response to treatment in half of the patients treated with these inhibitors.

Based on our data, levels of expression of the mutant form and activation of EGFR and its dimerizing partners varied significantly across MCPs established from the same tumor and affected response to treatment with different RTK inhibitors. Greater activation of mitogenic signaling molecules will most likely translate into increased proliferation rates as seen in resistant MCPs (e.g. higher KI67). Gu et al. have shown that *EGFR* mutant NSCLC whose tumors have low expression levels of Ki67 have significant longer overall survival compared to patients with high levels of expression of the proliferative marker when treated with TKIs [59]. Coupling NGS-based panels with clinically relevant assays (e.g. immunohistochemistry) suitable for measuring, for example, expression levels of mutant EGFR in patients’ samples or Ki67, may provide new opportunities for predicting long- or short-term responses to these targeted agents and for understanding clonal composition of NSCLCs in the clinic. The identification of signaling molecules utilized by MCPs that are less sensitive to treatment may also be critical for informing the development of combination treatments that will specifically benefit patients destined for rapid disease progression [60, 61].

As suggested by the unsupervised analysis in Fig. 5A, less responsive MCPs have broad activation of EGFR downstream signaling molecules belonging to the PI3K/AKT/mTOR pro-survival signaling axis in the absence of oncogenic mutations of members of the PIK3CA pathway, as these have not been described in the H1975 parental cell line [29]. A pan-cancer proteogenomic survey of the PI3K/AKT/mTOR pathway conducted on 11,219 tissue samples has shown that nearly one-fifth of tumors have high mTOR pathway activation that cannot be explained by the presence of underlying genomic alterations of the *PIK3CA* signaling axis [62]. While *PIK3CA* mutations are relatively rare events in NSCLCs, including in tumors harboring oncogenic mutations of the *EGFR* gene [63–67], activation of PI3K/AKT/mTOR signaling axis has been identified in 50–70% of tumors. This activation is often not attributable to underlying genomic alterations of members of this pathway [68–70]. Previous studies have suggested that in NSCLCs, increased Akt and mTOR phosphorylation is associated with poorer survival and drives resistance to EGFR and other RTK inhibitors [60, 71]. From our findings, we demonstrated that activation of this signaling axis can be highly heterogeneous even within the same tumor, and this heterogeneity defines, as expected, response to anti-EGFR treatment. Thus, the MCPs may become a preclinical instrumental tool for the early identification of molecular phenotypes that make subpopulations of cells intrinsically less sensitive to treatment. Tracing these biosignatures in patient samples may help understand clonal composition and predict short- and long-term mechanisms of resistance a tumor may utilize to overcome the effect of treatment.

As a tool for characterizing heterogeneous behaviors of coexisting subpopulations of cells, the MCPs are an instrumental model to functionally test heterogeneous dependency on specific molecular biosignatures within a tumor. Expanding the use of MCPs to co-culture or multicellular systems will provide new opportunities for understanding cellular and molecular dynamics driving clonal interactions and how cell-cell interactions shape the tumor microecology and therapeutic responses.

While our system offers unique opportunities for functionally assessing response to treatment across different MCPs within an individual tumor, a few limitations of the study need to be addressed. First, there is an inherent selection bias during the clonal expansion process, as less than 20% of seeded single cells successfully established MCPs. This relatively low rate of success is intrinsically linked to the ability of individual cells to survive and proliferate in the absence of neighboring cells. However, this inherent selection bias also allows us to directly characterize and functionally perturb cell subpopulations that have more aggressive phenotypes and thus are more likely to drive resistance and tumor progression in patients. Second, we did not conduct extensive DNA sequencing on the MCPs as part of this preliminary study, thus, we cannot fully attest whether multiple MCPs derived from the same clone. We expect to characterize these models in future studies to fully understand how the interplay of genetic variation, epigenetic regulation, and expression/activation of drug targets and downstream substrates modulates response to treatments in heterogeneous tumors. Nonetheless, the data presented in this work clearly demonstrate that MCPs established from a complex tumor have distinct phenotypic characteristics (morphology, signaling dynamics, response to treatment) when compared to each other and the parental line from which they are generated. To our knowledge, in vitro models able to capture this heterogeneity at the functional level are currently not available to the scientific community. However, they may become essential tools for linking heterogeneous behaviors within a tumor (e.g. response to treatment, transdifferentiation, invasion, etc.) to the underlying molecular events that specifically define these phenotypic traits. NSCLCs acquiring resistance to anti-EGFR treatment through genomic-independent events have been described as harder to treat in the clinic than those where resistance is driven by on- or off-target mutations [72]. As signal dynamics are the ultimate manifestation of transcriptional, translational, and post-translational reprogramming in cancer, approaches like the one proposed in this work may become highly relevant in the future to understand how phenotypic traits drive resistance to targeted compounds.

Third, by using the RPPA as our readout method, this work does not provide proteome-wide coverage. However, for this proof-of-concept study, we specifically designed an RPPA panel that encompasses signaling molecules known to be associated with response (or lack of) to anti-EGFR treatment. This approach allowed us to identify differences in the activation level of drug targets and downstream substrates of FDA-approved or experimental agents and directly test the effect of their inhibition. In line with what we observed when MCPs were treated with osimertinib, we detected differences in response rates across subpopulation of cells explained by the underpinning molecular profile of the MCPs. As we move forward with this work, we envision characterizing the MCPs using a multi-omic approach (e.g. proteo-transcriptomic and epigenetic analysis, etc.) to identify new off-target events and test their effects at the functional level. With therapeutics targeting epigenetic regulators currently undergoing intense preclinical and clinical evaluation and showing promise to overcome resistance in non-genomically driven tumors, understanding the role of epigenetic regulators in shaping intra-tumor heterogeneity and response to treatment may open new opportunities for devising combination treatments that specifically target subpopulations that are intrinsically or prone to resistance [72–74].

While comparing molecular profiles and response to anti-EGFR treatment across MCPs established by different cell lines would have strengthened the relevance of our biological observations, for this proof-of-concept study, we have specifically selected a cell line harboring two mutations of the EGFR gene known to be associated with response to anti-EGFR treatment. As shown by our data, even if both mutations were detected in all MCPs, responses varied greatly across cell subpopulations. However, MCPs with comparable responses to osimertinib (e.g. supersensitive vs. resistant MCPs) had similar molecular profiles as shown in the unsupervised clustering (Fig S9) and in the phylogenetic analysis (Fig. 5C). These findings provide an internal validation of how specific phenotypic traits (levels of expression of mutant EGFR, activation of AKT-mTOR signaling, etc.) within a heterogeneous tumor are associated with levels of sensitivity to treatment in NSCLCs.

Lastly, establishing MCPs from individual patients is not feasible, as the process is labor-intensive and time-consuming. Nonetheless, as in vitro testing is routinely incorporated in preclinical study, the use of MCPs in early testing may provide insights on mechanisms of resistance and response rates to new compounds across heterogeneous, but coexisting, cell subpopulations.

## Conclusions

Understanding and targeting tumor heterogeneity is still a major problem in oncology. While several studies have evaluated the degree of heterogeneity in tumors [75–78], translating genomic heterogeneity into solutions that can directly benefit patients has been limited. MCP-based models can provide important insights for testing the effect of new anticancer compounds across coexisting clones and for identifying biosignatures underpinning drug resistance uniquely attributable to clones that are intrinsically less responsive to treatment. These biosignatures may become instrumental for tracing clonal composition in clinical samples using emerging single cell technologies [79, 80] as well as spatial biology tools [81, 82]. Mapping these biosignatures at the tissue level will open new opportunities for predicting response to targeted treatments, and for devising single agents or combination therapies specifically designed to target the clonal composition of individual tumors.

### Electronic supplementary material

Below is the link to the electronic supplementary material.

Supplementary Material 1

## Data Availability

RPPA intensity values used for this analysis will be available after acceptance of the manuscript in Dataverse, an institutional data repository at George Mason University (https://dataverse.orc.gmu.edu).

## Abbreviations

BSA: Bovine serum albumin
EGFR: Epidermal growth factor receptor
EMT: Epithelial-to-mesenchymal transition
IC50: Half-maximal inhibitory concentration
MCP: Monoclonal cell population
NSCLC: Non-small cell lung cancer
PBS: Phosphate-buffered saline
PCR: Polymerase chain reaction
RPPA: Reverse phase protein microarray
RTK: Receptor tyrosine kinase
SEM: Standard error of the mean
TKI: Tyrosine kinase inhibitor
