# Supplementary material for: Signaling dynamics in coexisting monoclonal cell subpopulations unveil mechanisms of resistance to anti-cancer compounds
Source: Cell Commun Signal. 2024 Jul 26;22:377. doi: 10.1186/s12964-024-01742-3 (PMC11282632; doi:10.1186/s12964-024-01742-3)
Supplement: Supplementary file 1 — Supplementary Material 1 [file 12964_2024_1742_MOESM1_ESM.pdf]

Figure S1. Brightfield images and morphological classification of the 25 MCPs established from the H1975 cell line. M = mixed; C = cobblestone; E = elongated; S = syncytia-forming.

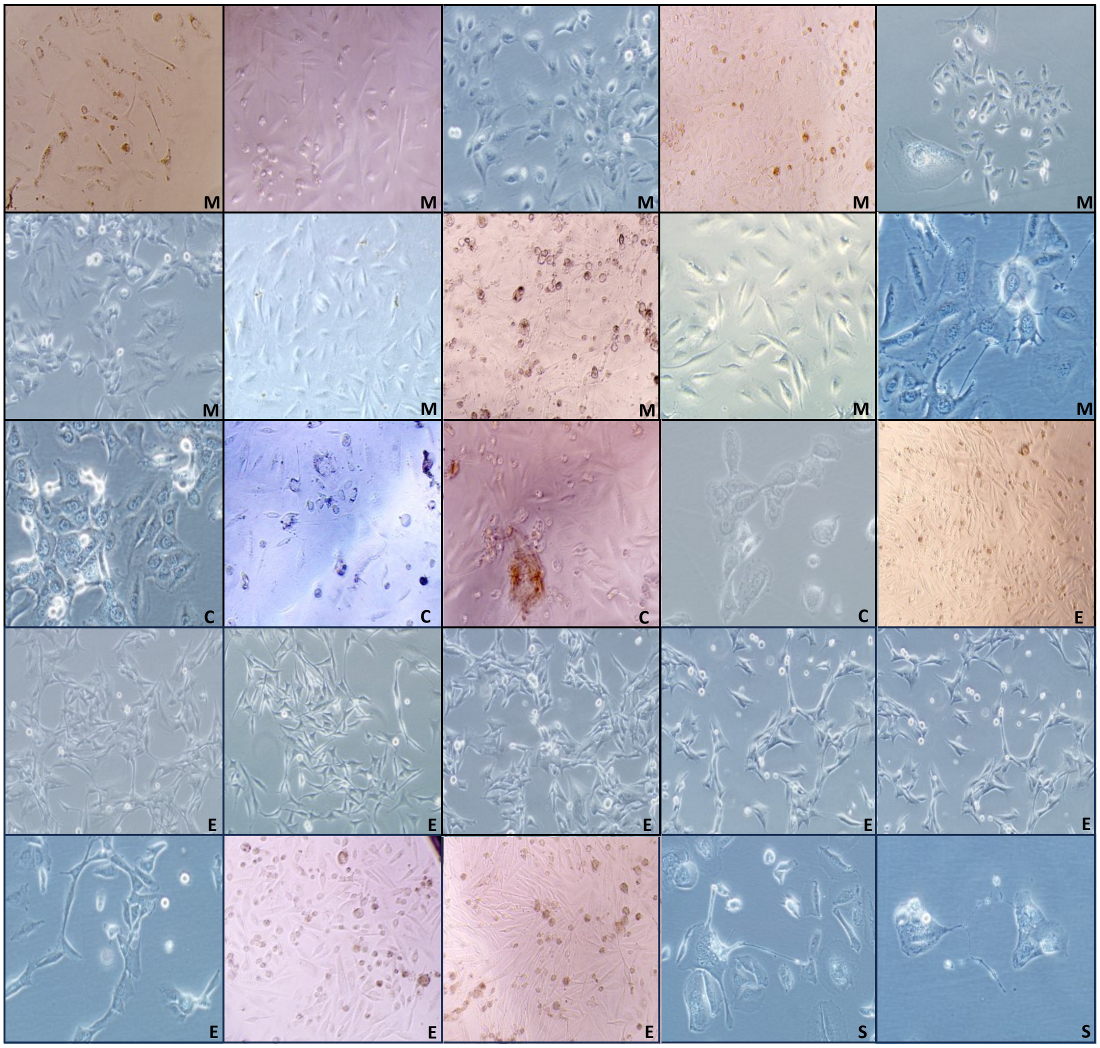

Figure S2. Brightfield images of spheroids established from 14 MCPs along with the morphological characteristics (mixed (m), cobblestone (c), syncytia-forming (s), and elongated (e)) of each clone when grown in monolayer.

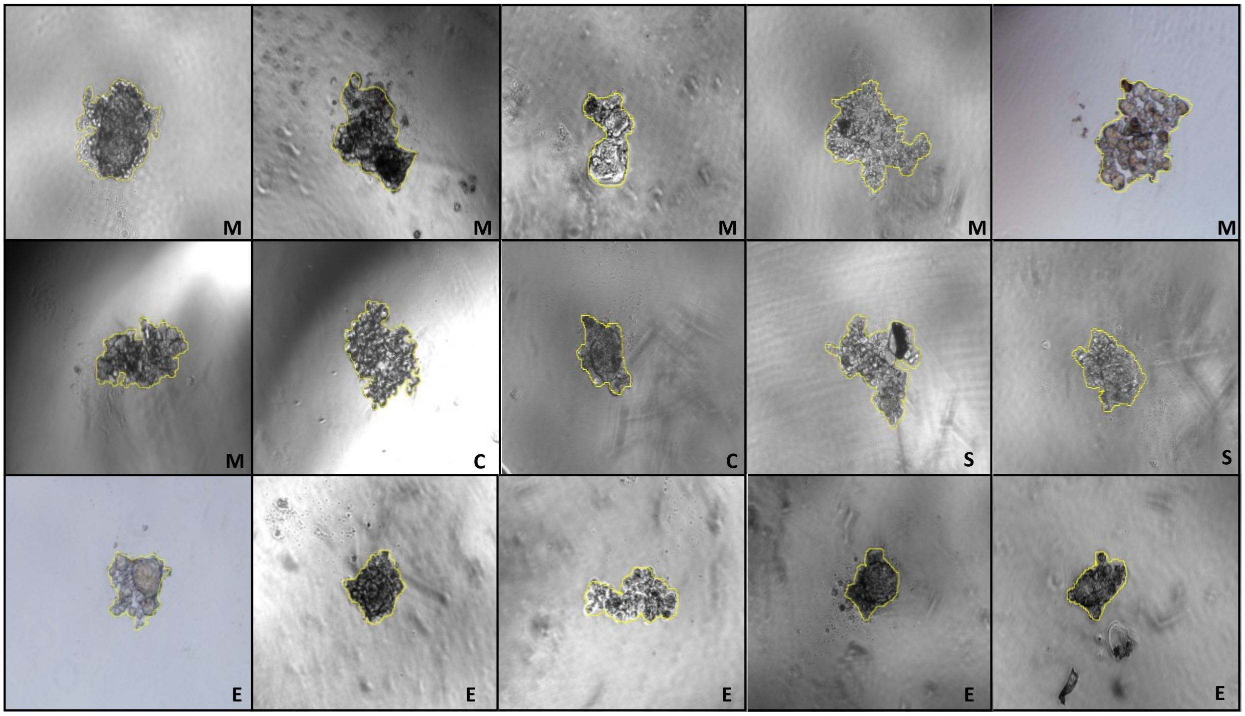

Figure S3. Dose-response curve of the H1975 parental cells treated with osimertinib for 72 hours. A non-linear regression, four parameters curve fit model was used to establish the IC50 value (700nM). Data were collected in technical (n=2) and experimental (n=4) replicates using two separate passages. Each point of the curve was normalized to the matching DMSO control and normalized values were rescaled on the untreated cells.

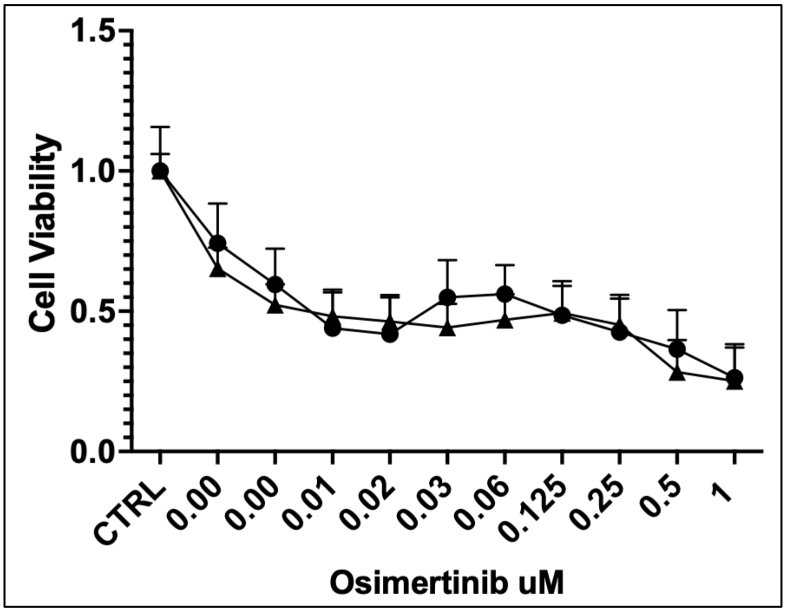

Figure S4. Selected examples of exon 20 and 21 of the EGFR gene sequencing electropherograms for the parental line, 22 MCPs, and the A549 cells used as negative control (Panel A). Unsupervised hierarchical clustering analysis of the 125 signaling molecules measured across the 25 MCPs (black), the parental line, and 14 NSCLC control cell lines (grey) with diverse genomic background (Panel B).

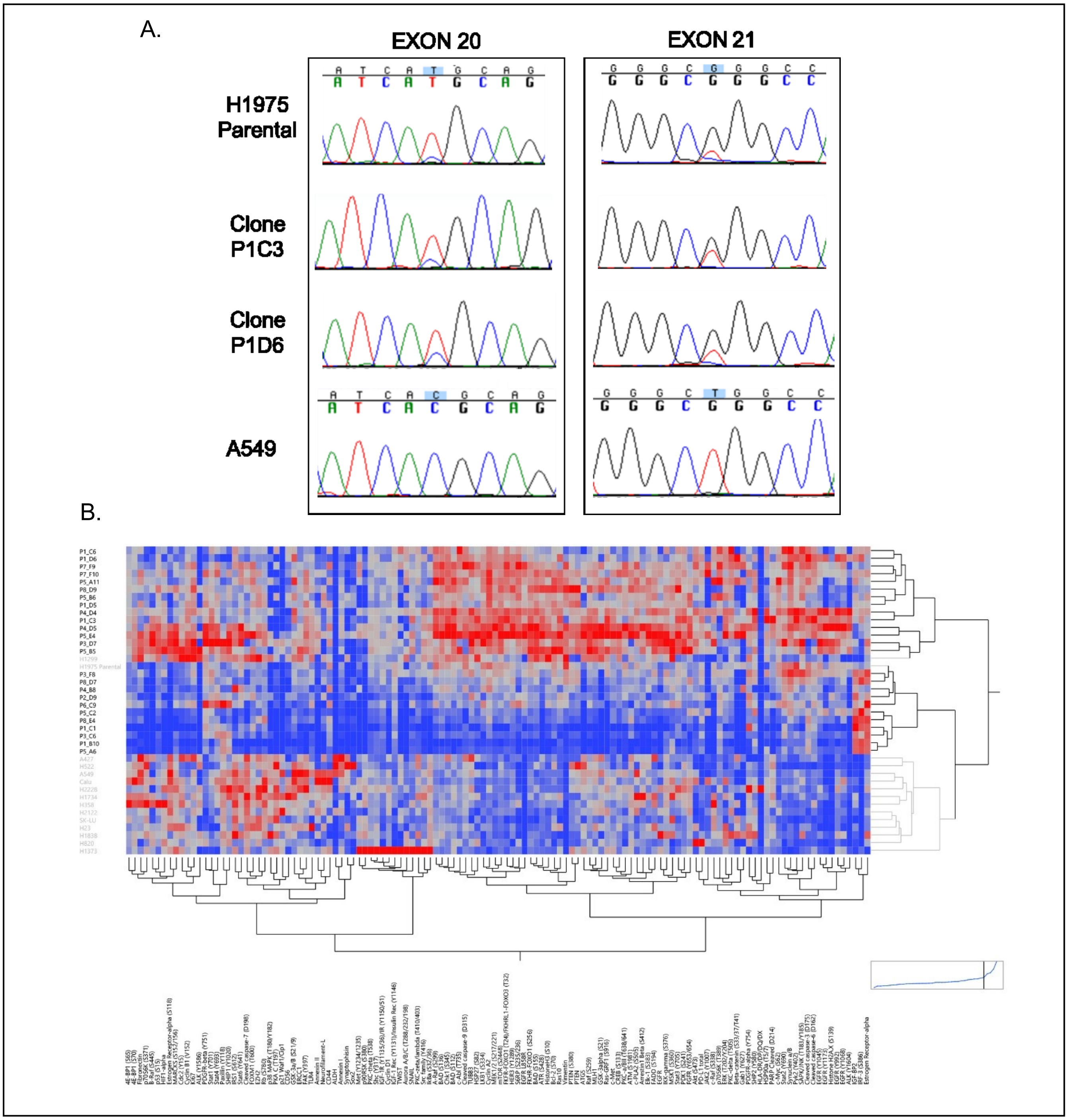

Figure S5. Examples of the single clone selection and expansion process for two MCPs from the isolation of individual cells through the establishment of individual cell lines with unique morphological features.

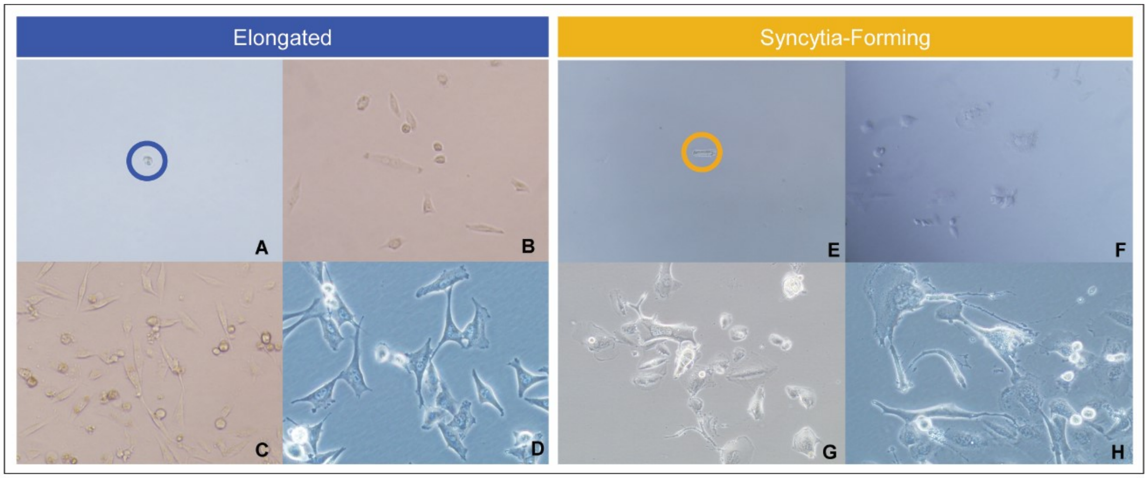

Figure S6. Correlation matrix capturing expression or activation levels of RTKs measured by RPPA. Strength and directionality of the correlations are captured by the circle dimension and color, respectively.

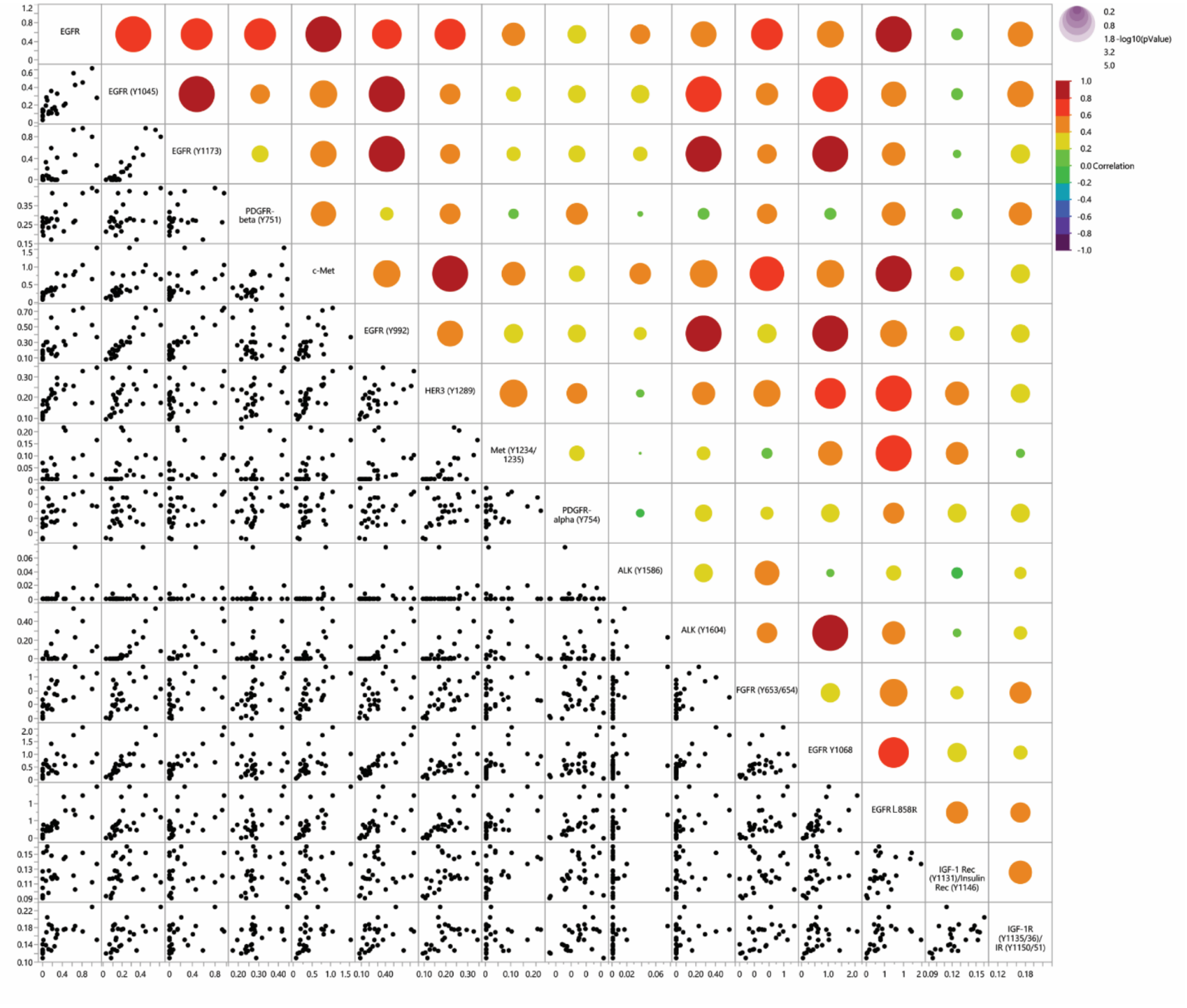

Figure S7. Unsupervised hierarchical clustering analysis of phosphorylated Akt, p70S6K, and C-Raf across MCPs and control lines. Samples are listed on the y-axis and are color-coded based on their response to osimertinib.

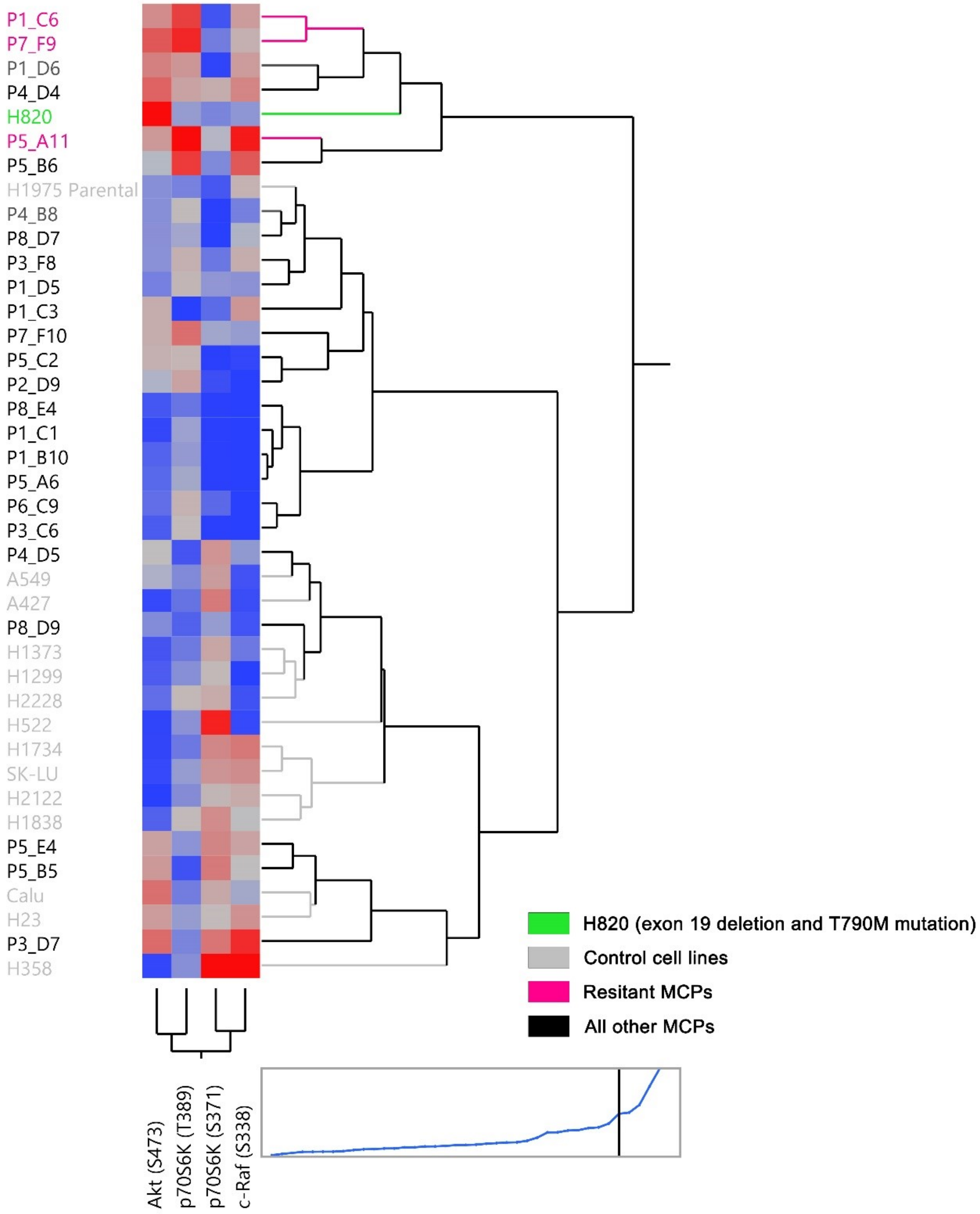

Figure S8. Unsupervised hierarchical clustering analysis of biological replicates for selected MCPs and the parental line for proteins bellowing to the MAPK and AKT-mTOR signaling pathway. Matched biological replicates are color-coordinated.

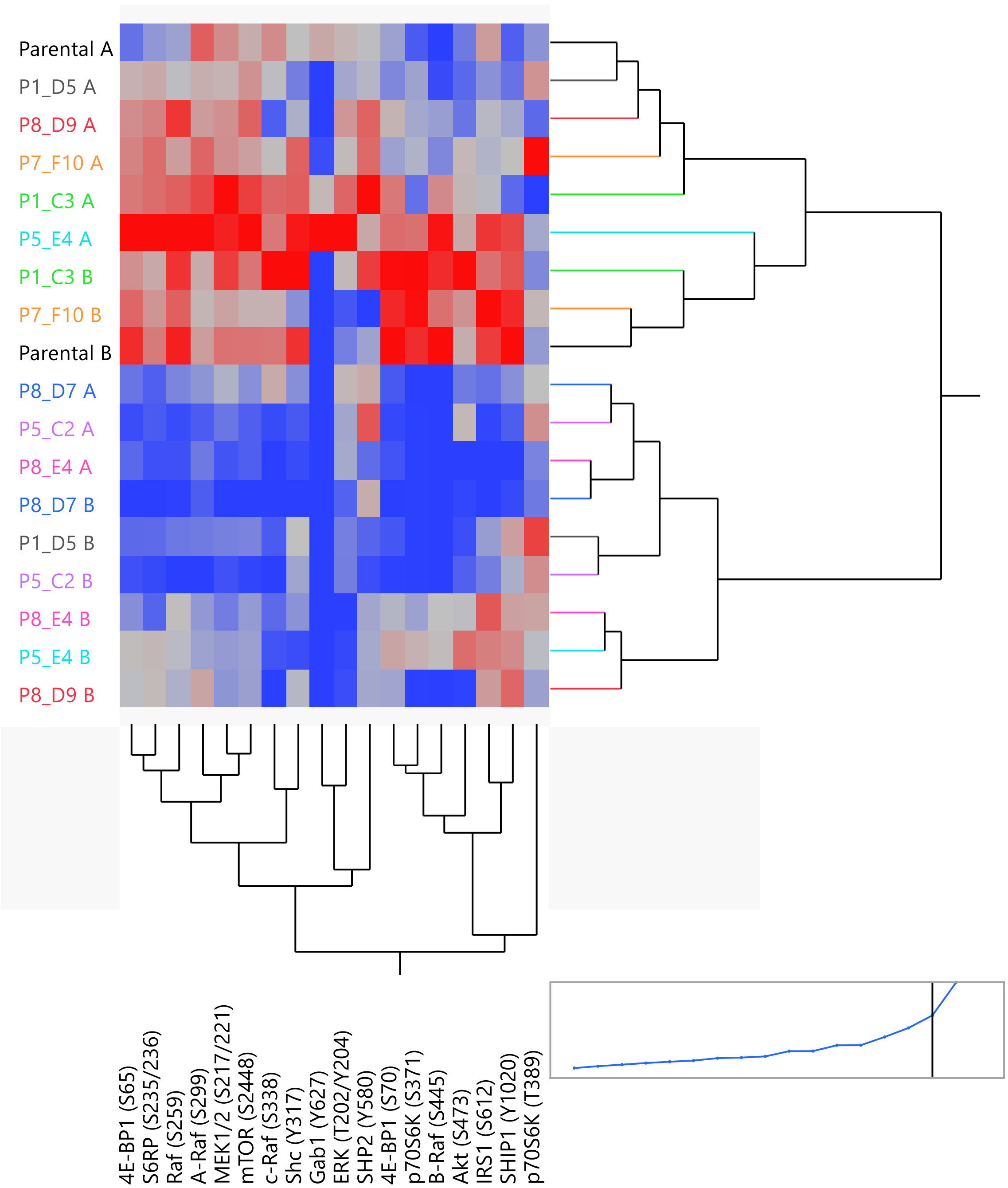

Figure S9. Unsupervised hierarchical clustering analysis of the 125 signaling molecules measured by RPPA across the 25 MCPs and parental line. Samples are listed on the y-axis and are color-coded based on their response to osimertinib.

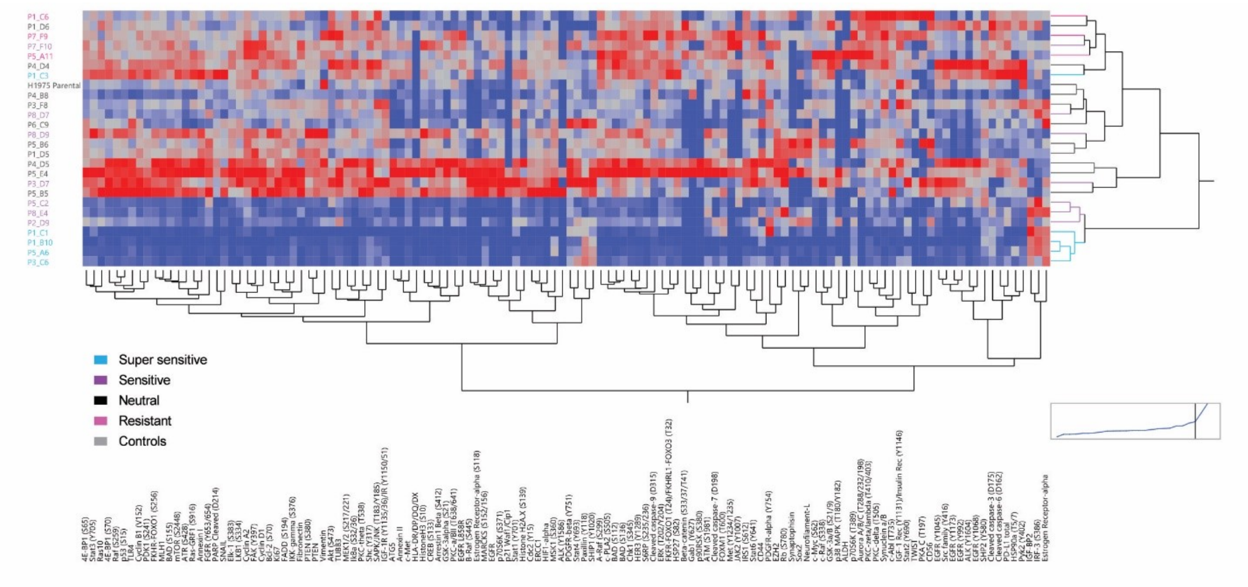

Figure S10. Unrooted phylogenetic neighbor joining tree depicting relatedness across models including MCPs and the parental line based on 33 signaling molecules including RTKs and downstream substrates belonging to the MAPK and AKT/mTOR signaling pathway. MCPs were color-coded based on their levels of response to treatment with osimertinib.

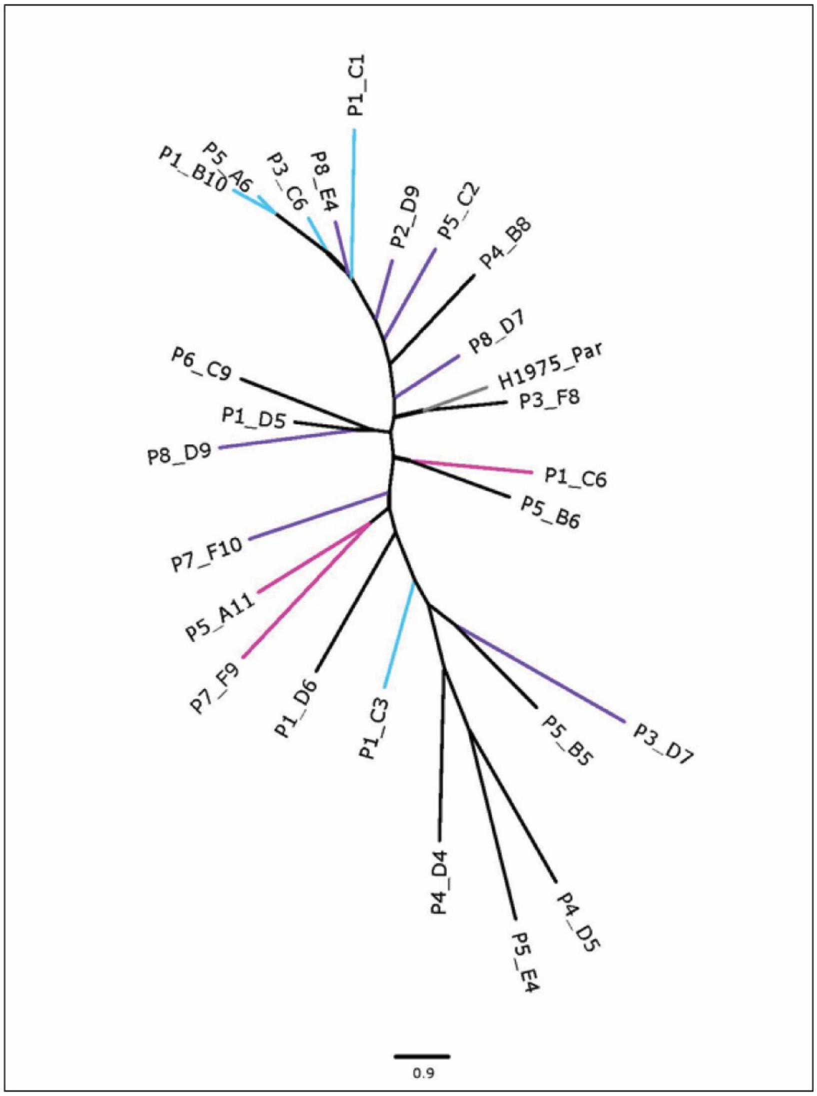

Figure S11. Unsupervised hierarchical clustering analysis of phosphorylated PDGFRs and markers of EMT across MCPs and control lines.

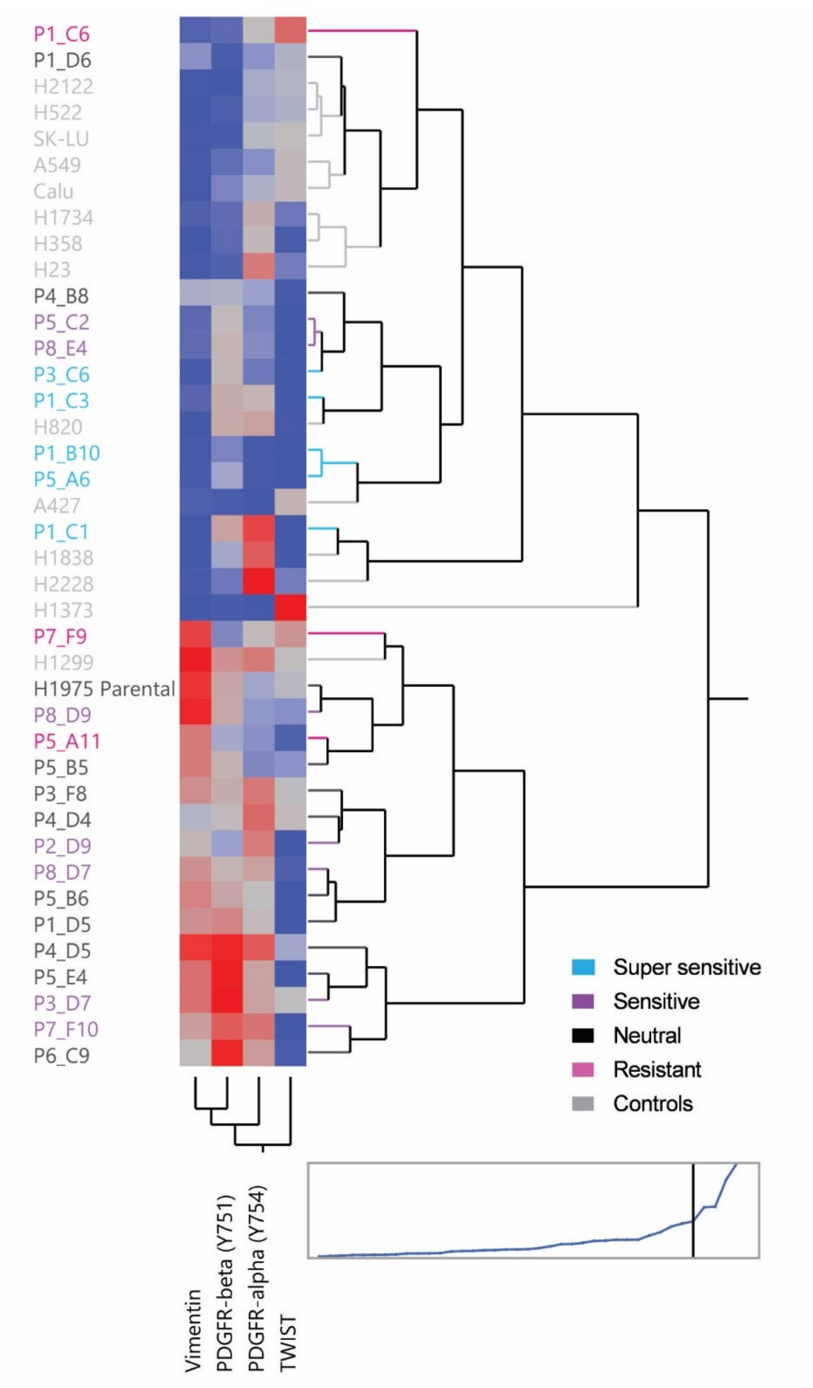

Table S1. Primer sequences used to amplify EGFR exons 20 and 21 by PCR.

EGFR Exon	Forward sequence	Reverse sequence
20	GAAACTCAAGATCGCATTCATGC	GCAAACTCTTGCTATCCCAGGAG
21	CAGCCATAAGTCCTCGACGTGG	CAGCCATAAGTCCTCGACGTGG

Table S2. List of antibodies used for the RPPA along with vendor, catalogue number, species (M: Mouse; R: Rabbit), dilution, and Kruskal–Wallis one-way analysis of variance p-values derived from the three-group comparisons across drug response classes (resistant, sensitive, and supersensitive). Significant p-values are bolded and italicized.

Antibody	Catalog number	Vendor	Dilution	Host	p value
4E-BP1 (S65)	9451	Cell Signaling	1:50	R	0.18
4E-BP1 (T70)	9455	Cell Signaling	1:200	R	0.15
AKT (S473) XP	4060	Cell Signaling	1:100	R	***0.03***
ALDH	611194	BD	1:1500	M	0.68
ALK (Y1604)	3341	Cell Signaling	1:50	R	0.79
Annexin I	610066	BD	1:5000	M	0.10
Annexin II	610068	BD	1:500	M	0.26
A-Raf (S299)	4431	Cell Signaling	1:50	R	***0.03***
Arrestin1 (Beta) (S412) [6-24]	2416	Cell Signaling	1:100	M	0.06
ATG5	2630	Cell Signaling	1:1000	R	0.41
ATM (S1981) [D6H9]	5883	Cell Signaling	1:50	R	0.19
ATR (S428)	2853	Cell Signaling	1:50	R	0.27
Aurora A (T288)/B (T232)/C (T198) [D13A11]	2914	Cell Signaling	1:50	R	***0.02***
BAD (S112)	9291	Cell Signaling	1:200	R	***0.05***
BAD (S136)	9295	Cell Signaling	1:50	R	***0.03***
BAD (S155)	9297	Cell Signaling	1:100	R	0.07
Bcl-2 (S70) [5H2]	2827	Cell Signaling	1:50	R	0.08
B-Raf (S445)	2696	Cell Signaling	1:50	R	0.42
c-Abl (T735)	2864	Cell Signaling	1:50	R	***0.02***
Caspase-3, cleaved (D175)	9661	Cell Signaling	1:50	R	0.09
Caspase-6, cleaved (D162)	9761	Cell Signaling	1:50	R	0.16
Caspase-7, cleaved (D198)	9491	Cell Signaling	1:100	R	0.11
Caspase-9, cleaved (D315)	9505	Cell Signaling	1:100	R	***0.03***
Catenin (beta) (S33/37/T41)	9561	Cell Signaling	1:100	R	0.23
CD44 [156-3C11]	3570	Cell Signaling	1:50	M	0.06
cdc2 (Y15) [10A11]	4539	Cell Signaling	1:50	R	0.18
Chk-1 (S345)	2341	Cell Signaling	1:50	R	0.18
c-Met	ab51067	Abcam	1:200	R	0.13
c-Myc (S62) [E1J4K]	13748	Cell Signaling	1:100	R	0.08
c-PLA2 (S505)	2831	Cell Signaling	1:1000	R	***0.02***
c-Raf (S338) [56A6]	9427	Cell Signaling	1:200	R	0.07
CREB (S133) [1B6]	9196	Cell Signaling	1:200	M	0.07
Cyclin A2 [BF683]	4656	Cell Signaling	1:50	M	***0.04***

Cyclin B1 [V152]	4135	Cell Signaling	1:200	M	0.13
Cyclin D1 [G124-326]	554180	BD	1:100	M	***0.04***
EGFR	2232	Cell Signaling	1:100	R	0.14
EGFR (Y1045)	2237	Cell Signaling	1:50	R	0.14
EGFR (Y1068)	2234	Cell Signaling	1:50	R	0.15
EGFR (Y1173)	44-794G	Thermo Fisher	1:100	R	0.34
EGFR (Y992)	2235	Cell Signaling	1:50	R	0.27
EGFR L858R	3197	Cell Signaling	1:100	R	***0.04***
Elk-1 (S383)	9181	Cell Signaling	1:100	R	***0.05***
ERCC1 (4F9)	UM500008	Origene	1:200	M	***0.03***
ERK (T202/Y204)	9101	Cell Signaling	1:1000	R	***0.05***
Estrogen Receptor alpha (S118) [16JR]	2511	Cell Signaling	1:1000	M	0.11
Estrogen Receptor α [D6R2W]	13258	Cell Signaling	1:200	R	0.11
EZH2 (D2C9) XP	5246	Cell Signaling	1:500	R	0.08
FADD (S194)	2781	Cell Signaling	1:100	R	0.09
FAK (Y397) [18]	611806	BD	1:50	M	0.11
FGF Receptor (Y653/654)	PA5-64626	Thermo Fisher	1:100	R	0.17
Fibronectin [IST-9]	ab6328	Abcam	1:100	M	***0.03***
FKHR-FOX01 (S256)	9461	Cell Signaling	1:100	R	0.16
FKHR-FOX01 (T24)/FKHRL1-FOX03 (T32)	9464	Cell Signaling	1:200	R	0.10
FOXM1 (T600)	14655	Cell Signaling	1:100	R	0.12
Gab1 (Y627)	3231	Cell Signaling	1:1000	R	***0.01***
GSK-3a/B (S21/9)	9331	Cell Signaling	1:100	R	0.09
GSK-3alpha (S21) (46H12)	9337	Cell Signaling	1:100	M	0.13
HER3 (Y1289) [21D3]	4791	Cell Signaling	1:200	R	0.13
HIF-1alpha [54]	610958	BD	1:50	M	0.25
Histone H2A.X (S139)	9718	Cell Signaling	1:50	R	0.15
Histone H3 (S10)	06-570	Upstate	1:200	R	0.11
HLA-DR/DP/DQ/DX (CR3/43)	sc-53302	Santa Cruz	1:50	M	0.29
HSP27 (S82)	2406	Cell Signaling	1:100	R	0.15
HSP90a (T5/7)	3488	Cell Signaling	1:100	R	0.75
IGF-1R (Y1131)/IR (Y1146)	3021	Cell Signaling	1:500	R	***0.04***
IGF-1R (Y1135/36)/IR (Y1150/51) [19H7]	3024	Cell Signaling	1:500	R	0.14
IGFBP2	3922	Cell Signaling	1:100	R	0.31
IkappaB-alpha (S32/36) [5A5]	9246	Cell Signaling	1:100	M	0.07
IKKγ (S376)	2689	Cell Signaling	1:50	R	0.39
IRF-3 (S386) [E7J8G]	37829	Cell Signaling	1:100	R	0.24
IRS-1 (S612)	2386	Cell Signaling	1:200	R	0.08
Jak2 (Y1007)	4406	Cell Signaling	1:200	R	0.51
Ki67 [MIB-1]	M7240	DAKO	1:100	M	***0.02***
LKB1 (S334)	3055	Cell Signaling	1:50	R	0.10

MARCKS (S152/156)	2741	Cell Signaling	1:200	R	0.50
MEK1/2 (S217/221)	9121	Cell Signaling	1:200	R	0.10
Met (Y1234/1235)	3126	Cell Signaling	1:200	R	***0.03***
MLH1 [4C9C7]	3515	Cell Signaling	1:500	M	0.14
MSK1 (S360)	9594	Cell Signaling	1:50	R	0.47
mTOR (S2448)	2971	Cell Signaling	1:100	R	0.16
NCAM1 (CD56) [E7X9M]	99746	Cell Signaling	1:100	R	0.19
Neurofilament-L [C28E10]	2837	Cell Signaling	1:100	R	0.11
p21 Waf1/Cip1 [DCS60]	2946	Cell Signaling	1:200	M	0.29
p38 MAPK (T180/Y182)	9211	Cell Signaling	1:100	R	0.77
p53 (S15)	9284	Cell Signaling	1:1000	R	0.14
p70 S6 Kinase (S371)	9208	Cell Signaling	1:50	R	0.19
p70 S6 Kinase (T389)	9205	Cell Signaling	1:100	R	***0.03***
p90RSK (S380)	9341	Cell Signaling	1:200	R	0.62
PARP, cleaved (D214)	9541	Cell Signaling	1:100	R	0.20
Paxillin (Y118)	2541	Cell Signaling	1:500	R	0.18
PDGF Receptor alpha (Y754) [23B2]	2992	Cell Signaling	1:500	R	0.46
PDGF Receptor beta (Y751)	3161	Cell Signaling	1:50	R	0.09
PDK1 (S241)	3061	Cell Signaling	1:200	R	0.18
PD-L1 [E1L3N] XP	13684	Cell Signaling	1:500	R	0.15
PKA C (T197)	4781	Cell Signaling	1:200	R	***0.03***
PKC a/BII (T638/641)	9375	Cell Signaling	1:100	R	***0.02***
PKC delta (T505)	9374	Cell Signaling	1:50	R	***0.02***
PKC theta (T538)	9377	Cell Signaling	1:100	R	0.24
PKC zeta/lambda (T410/403)	9378	Cell Signaling	1:50	R	***0.02***
PTEN	9552	Cell Signaling	1:50	R	0.06
PTEN (S380)	9551	Cell Signaling	1:500	R	0.09
Pyk2 (Y402)	3291	Cell Signaling	1:200	R	0.27
Raf (S259)	9421	Cell Signaling	1:100	R	0.11
Ras (RAS10)	05-516	Upstate	1:200	M	0.18
Ras-GRF1 (S916)	3321	Cell Signaling	1:50	R	0.17
Rb (S780)	3590	Cell Signaling	1:2000	R	***0.05***
S6 Ribosomal Protein (S235/236) [2F9]	4856	Cell Signaling	1:200	R	***0.05***
SAPK/JNK (T183/Y185)	9251	Cell Signaling	1:100	R	0.11
Shc (Y317)	2431	Cell Signaling	1:100	R	0.46
SHIP1 (Y1020)	3941	Cell Signaling	1:50	R	0.07
SHP2 (Y580) [D66F10]	5431	Cell Signaling	1:50	R	0.32
Snail [SN9H2]	4719	Cell Signaling	1:2000	Rat	0.50
Sox2	2748	Cell Signaling	1:100	R	0.49
Src Family (Y416)	2101	Cell Signaling	1:100	R	0.45
Stat1 (Y701) [D4A7]	7649	Cell Signaling	1:100	R	0.23

Stat2 (Y690)	4441	Cell Signaling	1:100	R	0.08
Stat3 (Y705) [D3A7]	9145	Cell Signaling	1:100	R	0.24
Stat4 (Y693)	5267	Cell Signaling	1:100	R	0.90
Stat6 (Y641)	9361	Cell Signaling	1:100	R	0.18
Synaptophisin	36406	Cell Signaling	1:100	R	0.13
Synuclein, a/B (Syn205)	2644	Cell Signaling	1:500	M	***0.01***
TLR4	sc-293072	Santa Cruz	1:100	M	0.18
Tubulin B III (TUBB3) [5H2]	TA500047	Origene	1:500	M	0.09
TWIST	sc-81417	Santa Cruz	1:100	M	***0.02***
Vimentin [D21H3]	5741	Cell Signaling	1:200	R	0.01

Table S3. Drug sensitivity scores for all MCPs when treated with osimertinib at the IC50 value of the parental line (700 nM). Scores are shown after normalization to the matched DMSO control and normalization to the parental line.

MCP	Drug score (osimertinib)
P1_C6	1.19
P7_F9	1.35
P1_D6	1.05
H1975 Parental	1
P4_B8	1.04
P5_A11	1.29
P3_F8	1.09
P4_D4	1.04
P4_D5	1.06
P1_C3	0.49
P7_F10	0.59
P5_E4	0.94
P8_D7	0.59
P5_C2	0.63
P8_D9	0.65
P5_B6	1.07
P1_D5	0.86
P8_E4	0.68
P2_D9	0.6
P1_C1	0.37
P6_C9	1.1
P1_B10	0.25
P5_A6	0.42
P3_C6	0.46
P3_D7	0.75
P5_B5	0.87
